# Human Gingival Fibroblasts Display a Non-Fibrotic Phenotype Distinct from Skin Fibroblasts in Three-Dimensional Cultures

**DOI:** 10.1371/journal.pone.0090715

**Published:** 2014-03-07

**Authors:** Wesley Mah, Guoqiao Jiang, Dylan Olver, Godwin Cheung, Ben Kim, Hannu Larjava, Lari Häkkinen

**Affiliations:** Department of Oral Biological and Medical Sciences, Faculty of Dentistry, University of British Columbia, Vancouver, Canada; University of Bergen, Norway

## Abstract

Scar formation following skin injury can be a major psychosocial and physiological problem. However, the mechanisms of scar formation are still not completely understood. Previous studies have shown that wound healing in oral mucosa is faster, associates with a reduced inflammatory response and results to significantly reduced scar formation compared with skin wounds. In the present study, we hypothesized that oral mucosal fibroblasts from human gingiva are inherently distinct from fibroblasts from breast and abdominal skin, two areas prone to excessive scar formation, which may contribute to the preferential wound healing outcome in gingiva. To this end, we compared the phenotype of human gingival and skin fibroblasts cultured in *in vivo*-like three-dimensional (3D) cultures that mimic the cells' natural extracellular matrix (ECM) niche. To establish 3D cultures, five parallel fibroblast lines from human gingiva (GFBLs) and breast skin (SFBLs) were seeded in high density, and cultured for up to 21 days in serum and ascorbic acid containing medium to induce expression of wound-healing transcriptome and ECM deposition. Cell proliferation, morphology, phenotype and expression of wound healing and scar related genes were analyzed by real-time RT-PCR, Western blotting and immunocytochemical methods. The expression of a set of genes was also studied in three parallel lines of human abdominal SFBLs. Findings showed that GFBLs displayed morphologically distinct organization of the 3D cultures and proliferated faster than SFBLs. GFBLs expressed elevated levels of molecules involved in regulation of inflammation and ECM remodeling (MMPs) while SFBLs showed significantly higher expression of TGF-β signaling, ECM and myofibroblast and cell contractility-related genes. Thus, GFBLs display an inherent phenotype conducive for fast resolution of inflammation and ECM remodeling, characteristic for scar-free wound healing, while SFBLs have a profibrotic, scar-prone phenotype.

## Introduction

Scar formation is a common yet unwanted wound healing outcome in skin. Recent estimates include that in developed countries, at least 100 million patients develop scars each year from surgical procedures alone. Scars vary from fine lines to excessive accumulation of scar tissue that can be esthetically unpleasing and cause psychological distress and morbidity, including pain and contracture, and limit functions such as joint mobility. Currently there is no clinically predictable and effective treatment to eliminate formation of scars emphasizing the need to better understand the biological processes that underlie these conditions [1], [2].

The hallmark of scar formation is excessive accumulation of abnormally organized type I collagen-rich extracellular matrix (ECM) during the remodeling stage of wound healing. It is likely caused by factors that derail the wound healing process at the earlier stages of wound healing. Key factors that have been associated with scar formation include age and sex of the individual, location, size and depth of the wound, increased and prolonged inflammatory reaction, and excess activity of profibrotic growth factors, including TGF-β1 [2], [3]. Ultimately, TGF-β1 and other factors may perturb functions of fibroblasts, cells responsible for ECM production and remodeling. In addition, they may promote recruitment, survival or accumulation of scar prone fibroblast subpopulations that deposit the excessive ECM [4]. The importance of fibroblast phenotype in scar formation is supported by findings showing that, for instance, scarless wound healing in the first and second trimester fetal skin associates with distinct phenotype of fibroblasts [3], [5]. In addition, cultured scar-derived fibroblasts are phenotypically different from normal skin fibroblasts [3], [5]. Moreover, a higher incidence of scar formation in deep than shallow dermal wounds corresponds to deep dermal fibroblasts phenotype that is scar-prone and distinct from superficial dermal cells [6]. Scars may also display persistence of active myofibroblasts subpopulations [7].

Studies using various human, pig and murine models have shown that wound healing in different parts of oral mucosa is markedly faster, and in gingiva results to significantly reduced clinical and histological scar formation, compared to similar wounds in skin. The reason for the preferential oral mucosal wound healing may in part depend on the milder and shorter inflammatory response in oral mucosa [5], [8]–[10]. However, other factors are also likely involved. For instance, the ECM niche created by fibroblasts, a key factor determining cell functions within tissues, appears different in oral mucosa and skin [5], [11]. In addition, experiments with cultured cells have suggested that oral mucosal fibroblasts have also some other properties that are distinct from adult skin fibroblasts, but similar to fetal skin cells [5], [12]. Fibroblasts from skin and oral mucosa have also distinct gene expression profiles that correlate with their different embryonic patterning-related HOX gene expression [13]–[16]. This may depend on the different embryonic origin of gingival and skin connective tissue cells, the former being mostly from the neural crest and the latter from the mesoderm [11], [17]. Thus, gingival fibroblasts may be phenotypically distinct from skin cells, and this may in part underlie the fast and relatively scarless wound healing response in oral mucosa. However, little is known about the phenotype of these cells in relation to mechanisms of wound healing and scar formation.

Scar formation is an outcome of a complex process of wound healing that is regulated by multiple interactions of various inflammatory, tissue resident and progenitor cells in a time-dependent manner. It also involves dynamic interactions of cells with a three-dimensional (3D) ECM niche composed of various cytokines, growth factors and ECM molecules [14]. Therefore, assessing fibroblasts in standard 2D monolayer cultures where cells attach to a planar tissue culture plastic surface only from the basal aspect of cell membrane does not well mimic the situation *in vivo*. A more natural approach is a culture model where cells generate and become embedded in their own 3D ECM niche. In this model, the organization and composition of the 3D ECM is *in vivo*-like and cells interact with it on all aspects of the cell surface using similar cell adhesion receptors found *in vivo* [18]–[20]. The 3D cultures also mimic the tissue niche functionally. For instance, they retain stem or tumor stromal cell phenotype better than traditional 2D cultures [21], [22]. Therefore, in the present study, the 3D culture model was used to compare the phenotype of human oral mucosal fibroblasts from attached gingiva, tissue characterized by little scar formation, with fibroblasts from human breast and abdominal skin, two areas of skin prone to excessive scar formation [23], [24]. The findings showed that gingival fibroblasts proliferated faster and expressed significantly higher levels of molecules that are involved in modulation of inflammation and ECM remodeling (MMPs), while skin cells showed significantly elevated expression of TGF-β signaling, ECM, myofibroblast and cell contractility-related genes. Thus, gingival fibroblasts display an inherent phenotype conducive for fast resolution of inflammation and ECM remodeling, typical to scarless wound healing, while skin fibroblasts have a profibrotic, scar-prone phenotype.

## Materials and Methods

### Cells

Dermal fibroblasts (SFBLs) from clinically healthy human breast (five lines from different donors) and abdominal skin (three lines from different donors) were obtained from PromoCell (Heidelberg, Germany). Five gingival fibroblast lines (GFBLs) were isolated as previously described [25] from clinically healthy attached gingiva from healthy human donors (Table 1). Cells were maintained at 37°C and 5% CO_2_ in Dulbecco's Modified Eagle's medium (DMEM), supplemented with 10% fetal bovine serum (FBS) and 1% antibiotic/antimycotic (Gibco Life Technologies, Inc., Grand Island, NY, USA). Cells were routinely seeded for experiments when they reached about 95% confluence. Experiments were performed at passages 5 to 11.

**Table 1:** List of the cell lines used.

Cell line name	Origin	Sex	Age (years)
GFBL-DC	Attached gingiva	Male	41
GFBL-OL	Attached gingiva	Male	30
GFBL-HN	Attached gingiva	Female	18
GFBL-DW	Attached gingiva	Female	30
GFBL-IE	Attached gingiva	Male	26
SFBL-2-C	Caucasian breast dermis	Female	40
SFBL-1-2	Caucasian breast dermis	Female	44
SFBL-4-1	Caucasian breast dermis	Female	41
SFBL-302	Caucasian breast dermis	Female	38
SFBL-406	Caucasian breast dermis	Female	35
SFBL-35	Caucasian abdominal skin	Female	38
SFBL-03	Caucasian abdominal skin	Female	35
SFBL-20	Caucasian abdominal skin	Female	34

### Ethics Statement

Gingival tissue donors provided a written informed consent, and procedures were reviewed and approved by the Office of Research Ethics of the University of British Columbia.

### Three-dimensional (3D) cell culture

GFBLs and breast and abdominal SFBLs were seeded at high density (5×10^4^ cell/cm^2^) on plastic culture plates in DMEM, supplemented with 10% FBS, 1% antibiotic/antimycotic and 50 µg/ml of ascorbic acid [26]. Cells were allowed to grow and deposit an *in vivo-like* 3D ECM up to 21 days, with medium change three times per week.

### Cell proliferation assays

Proliferation of GFBLs and breast SFBLs was studied in both low (starting density 1.6×10^4^ cell/cm^2^) and high (5×10^4^ cells/cm^2^) density conditions. For the low-density condition, three parallel GFBL and breast SFBL lines were seeded in 96-well plates in six replicates and cell numbers were recorded at day 1, 3, 6 and 8 post-seeding, using a tetrazolium-based colorimetric assay (MTT assay; Promega, Madison, WI, USA). To assess cell numbers at high density conditions, cells were seeded and maintained as described above for generation of 3D cell cultures for 3, 7, 10 and 14 days. Total RNA was extracted using NucleoSpin RNA II kit (Macherey-Nagel, Bethlehem, PA, USA) and quantitated by spectrophotometry (GeneQuant, LKB Biochrom, Ltd, Cambridge, UK) as a measurement of cell numbers. The experiments were repeated three times.

### Immunostaining

For immunostaining, GFBL and breast SFBL 3D cultures were generated on gelatin-coated glass coverslips [27]. Briefly, the coverslips were incubated in 0.2% gelatin in phosphate-buffered saline (PBS) at 37°C for 1 h. After rinsing with PBS, coverslips were incubated in 1% glutaraldehyde at room temperature for 30 min, then washed with PBS, followed by incubation with DMEM at 37°C for 30 min. Coverslips were then washed with PBS and stored at 4°C or used immediately. To generate 3D cell culture, three GFBL and breast SFBL lines were cultured on the coverslips as described above. At day 7 post-seeding, the cultures were fixed with 4% formaldehyde at room temperature for 20 min and permeabilized using 0.5% Triton X-100 in PBS for 4 min. All samples were then blocked with PBS containing Ca^2+^ and Mg^2+^ (PBS+), BSA (10 mg/ml) and glycine (1 mg/ml) at room temperature for 30 min, followed by an incubation with the primary antibody (Table S1) diluted in PBS containing BSA (1 mg/ml) in a humidified chamber at 4°C overnight. The samples were then washed with PBS containing BSA (1 mg/ml) and 0.01% Triton X-100, and incubated with an appropriate Alexa-conjugated secondary antibody (1∶100 dilution; Alexa 488/594; Molecular Probes Inc., Eugene, OR, USA) at room temperature for 1 h. Nuclei were then stained with 300 nM DAPI (Molecular Probes Inc.) in PBS for 5 min. Samples were mounted with Immuno-mount solution (Thermo Scientific, Pittsburgh, PA, USA), examined using an Axioplan II Fluorescent microscope (Carl Zeiss Inc., Jena, Germany), and images captured using Northern Eclipse software (Empix Imaging, Mississauga, ON, Canada).

### Real-time RT-PCR

Total RNA was extracted from 3D cultures using NucleoSpin RNA II kit and treated with rDNase according to the manufacturer's protocol (Macherey-Nagel). Briefly, cells were washed once with PBS and lysed with RA1 buffer containing 1% beta-mercaptoethanol at room temperature for 3–5 min. The lysate was filtrated through NucleoSpin Filter at 11,000×g for 1 min. Supernatants were mixed with equal volume of 70% ethanol and the mixture was centrifuged in the NucleoSpin RNA II Column at 11,000×g for 1 min. Samples were desalted with MDB buffer, followed by incubation with rDNase (10 U) at room temperature for 15 min. Samples were then washed with RA2 and RA3 buffer and total RNA was eluted from the column with RNase/DNase-free water. Total RNA concentration and purity was measured by RNA/DNA Calculator (GeneQuant Pro, Amersham Biosciences, Little Chalfont, Buckinghamshire, UK). RNA integrity was assessed by electrophoresis using a denaturing agarose gel containing formaldehyde, followed by staining of RNA with 0.5 µg/ml of ethidium bromide in 0.1 M ammonium acetate for 30 min. Gels were assessed for integrity of 18S and 28S rRNAs bands (1.9 kb and 5 kb, respectively). Samples with 1.8 to 2.0 of OD260/280 ratio, and approximately 2.1 ratio of 28S/18S rRNA, were used for the study. cDNA was synthesized using iScript Select cDNA Synthesis Kit (Bio-Rad) according to the manufacturer's instructions. Briefly, 1.0 µg of total RNA was reverse transcribed by adding 4 µl of 5× reaction buffer, 2 µl of random primers and 1 µl reverse transcriptase and nuclease-free water for a final volume of 20 µl. The cDNA was synthesized using Mastercycler gradient 5331 Reverse-Transcriptase PCR Instrument (Eppendorf AG, Hamburg, Germany) using the following program: 1 cycle at 25°C for 5 min, 1 cycle at 42°C for 30 min and 85°C for 5 min to heat-inactivate the reverse transcriptase. The primers used for real-time PCR are listed in Table S2. When possible, all primers were designed on the boundaries of exons, and analyzed by BLASTn software (http://blast.ncbi.nlm.nih.gov/Blast.cgi) for their specificity. The primers were designed to yield a target sequence that was 60–150 base pairs long with a GC content between 40–70%. Efficiency of the target amplification was optimized (which includes annealing temperature, primer and sample concentration) up to 95% for each primer set using a 10-fold dilution series of cDNA while standard curves were made. For the reaction, cDNA from each sample was diluted to a concentration (2 ng/μl) such that the Ct values were well within the range of their standard curves, and 5 µl of diluted cDNA was mixed with 10 µl of 2 X iQ SYBR Green I Supermix (Bio-Rad), and 5 pmoles of primers, for a final volume of 20 µl. Real-time PCR amplification was performed on the CFX96 System (Bio-Rad) using the following program: 1 cycle at 94°C for 3 min 35 cycles at 94°C for 10 s, 58°C for 20 s, and reaction completion with reading plate and a melt curve analysis from 65°C to 95°C, 5 s for each 0.5°C.

Amplification reactions were conducted for target genes with ubiquitin C (UBC), glyceraldehydes-3-phosphate dehydrogenase (GAPDH), hypoxanthine phosphoribosyltransferase I (Hprt1), 18s rRNA and asparagine-linked glycosylation 9 (ALG9) as reference genes. For a given experiment, at least two reference genes with a M-value below 0.5 were chosen [28]. Non-transcribed RNA samples were used as a negative control. The PCR reactions were performed in triplicate for each sample. The data was analyzed and is presented based on the comparative Ct method (CFX Manager Software Version 2.1, Bio-Rad). Gene expression analysis was performed using five parallel GFBL and breast SFBL lines, and three abdominal SFBL lines (Table 1). For GFBLs and breast SFBLs, the experiment was repeated at least three times, and for abdominal SFBLs twice.

### Collection of conditioned medium

GFBLs and breast SFBLs were grown in 3D cultures as described above, and at day 6 post-seeding culture medium was replaced with serum-free medium. After 48 h, the conditioned medium (CM) was collected and Complete Protease Inhibitor Cocktail (Roche Diagnostics, Manheim, Germany) was added. Samples were stored at 4°C until use. For Western blotting analysis, CM samples were concentrated (30–40 times) by centrifugation using Centrifugal Filter Units (Millipore, Bedford, MA, USA).

### Quantification of collagen abundance in conditioned medium

Total collagen content was determined by using Sircol Collagen Assay Kit (Biocolor Ltd., Carrickfergus, Northern Ireland, U.K.) according to the manufacturer's instructions. Briefly, equal volumes of CM were incubated with Isolation and Concentration Reagent at 4°C overnight. Samples were then centrifuged at 12,000 rpm for 10 min using a microcentrifuge, and Sircol Dye Reagent was then added to the pellet and incubated with shaking for 30 min. Samples were centrifuged as above to allow packing of the collagen-dye complex. The supernatant was discarded and cold Acid-Salt Wash Reagent was added without disrupting the pellet. Samples were centrifuged as above, the supernatant was discarded and Alkali Reagent was added to the pellet. Samples were vortexed and then measured at 595 nm using a spectrophotometer (Bio-Rad). Total collagen concentration was estimated using the Collagen Standard (Biocolor Ltd.). The experiment was performed using five parallel GFBL and breast SFBL cell lines.

### Quantification of total sulphated glycosaminoglycans in conditioned medium

Total sulphated glycosaminoglycans (GAGs) were determined using Blyscan Sulphated GAG Assay Kit (Biocolor Ltd.) according to the manufacturer's instructions. Briefly, equal volumes of CM samples were incubated with Blyscan Dye Reagent for 30 min and then centrifuged at 12,000 rpm for 10 min as above. The pellet was incubated with the Dissociation Reagent for 10 min and color reaction was quantitated at 655 nm using a spectrophotometer (Bio-Rad). Total amount of sulphated GAGs was estimated relative the Glycosaminoglycan Standard (Biocolor Ltd.). The experiment was performed in duplicate for five GFBL and breast SFBL lines.

### Quantification of total protein deposited in the cell-derived 3D ECM

Fibroblasts were grown in 3D culture for 3, 7, 10, and 14 days and cell-free ECM prepared as described previously [26]. Briefly, cultures were washed once with PBS and incubated with a cell extraction buffer composed of 0.5% Triton X-100 and 20 mM NH_4_OH in PBS (pH = 8.0) at 37°C for 5–10 min to remove cells. PBS was then added to dilute the extraction buffer 2-fold, and cultures were incubated at 4°C overnight. The cell-free 3D ECM was washed with PBS, treated with 10 U/ml of DNase (Roche Diagnostics, Indianapolis, IN, USA) at 37°C for 30 min, solubilized in SDS sample buffer (2% SDS, 0.005% bromophenol blue, 10% glycerol in 50 mM Tris-HCl; pH = 6.8) and collected using a rubber policeman. Total protein content was measured using Bio-Rad DC protein assay reagent and a spectrophotometer at 655 nm (Bio-Rad; model 3550 microplate reader) according to the manufacturer's instructions. Five GFBL and breast SFBL lines were used for the analysis, and the experiment was repeated three times.

### Preparation of cell lysates for Western blotting

GFBLs and breast SFBLs were grown in 3D cultures for 7 days, as described above. Cells were then washed with ice-cold PBS and lysed with 20 mM MOPS, 2 mM EGTA, 5 mM EDTA, 1% Triton X-100 containing Complete Protease Inhibitor Cocktail (pH = 7.2; Roche Diagnostics). Lysates were collected using a rubber policeman, sonicated on ice and filtered through a NucleoSpin Filter (Macherey-Nagel) by centrifugation with 5,000 g for 1 min. For α-SMA analysis, fibroblasts in 3D cultures were lysed in SDS sample buffer and collected using a rubber policeman.

### Western blotting

Total protein concentration in CM and cell/ECM lysates was determined using the DC Protein Assay (Bio-Rad). Equal amount of protein of each sample was solubilized in SDS sample buffer containing 2-mercaptoethanol (5%) and separated in 7.5–12% SDS-polyacrylamide gel electrophoresis. The proteins were then transferred onto a Hybond-ECL nitrocellulose membrane (Amersham Biosciences) at 4°C overnight. The nonspecific binding sites were blocked by incubating the membranes in Odyssey Blocking Buffer (LI-COR Biosciences; Lincoln, NE, USA) at room temperature for 1 h, followed by incubation with the primary antibody (Table S1) in Odyssey Blocking Buffer, containing 0.1% Tween-20 at 4°C overnight. After washing with TBS containing 0.1% Tween-20 (TBS-T), the membranes were incubated with an appropriate species-specific secondary antibody conjugated with IRdye (1∶10,000; LI-COR Biosciences) in Odyssey Blocking Buffer, containing 0.1% Tween-20 and 0.01% SDS at room temperature in the dark for 1 h. After washing with TBS-T (4 times for 5 min each), the blots were washed in TBS for 5 min and air-dried. The blots were detected using the LI-COR Odyssey Infrared Imaging system (LI-COR Biosciences). The results were quantified using the Odyssey application software version 3.0.

To confirm the identity of different molecular weight bands obtained in Western blots of small leucine-rich proteoglycans, a set of CM and cell/ECM samples was digested with 0.1 U/ml of keratanase II (Seikagaku Biobusiness Corp., Tokyo, Japan) in 50 mM Tris-HCl (pH = 7.4), 1 U/ml of chondroitinase ABC (Sigma-Aldrich, St. Louis, MO, USA) in 50 mM acetic acid and 30 mM of Tris-HCl (pH = 8.0) at 4°C overnight or with both enzymes consecutively (24 h incubation with chondroitinase ABC followed by 24 h incubation with keratanase II) before electrophoresis and Western blotting (data not shown).

To identify latent and active MMPs, a set of CM and cell/ECM samples were treated with or without p-aminophenylmercury acetate (APMA; 1.0 mM, pH = 7.4; Sigma-Aldrich) at 37°C for 4 h to activate latent enzymes [29] prior to gel electrophoresis and Western blotting (data not shown).

### Quantification of myofibroblasts

Three parallel GFBL and breast SFBL lines were analyzed for the presence of cells with α-SMA-rich cytoskeleton by immunostaining. Cells at early (P5–7) and late (P8–11) were seeded in high density on coverslips in their normal growth medium and cultured for 48 h followed by fixation with 4% formaldehyde at room temperature for 20 min before immunostaining of α-SMA (Table S1) as described above. Samples were examined using Axioplan II Fluorescent microscope (Carl Zeiss Inc., Jena, Germany) and digital images were obtained from randomly selected areas using x40 objective. Proportion of cells with myofibroblast-like α-SMA-rich cytoskeletal stress fibers was calculated relative to total number of cells identified by nuclear DAPI staining.

### Assessment of cell senescence

Three parallel GFBL and breast SFBL lines at early (P5–7) and late (P8–11) passage were seeded at high density on coverslips coated with gelatin and cultured in their normal growth medium for 48 h. Cells were then fixed with 2% formaldehyde/0.2% glutaraldehyde at room temperature for 15 min. Senescence-associated β-galactosidase staining was performed according to the manufacturer's instructions (Cell Signaling Technologies, Danvers, MA, USA). Briefly, coverslips were incubated with β-Galactosidase Staining Solution (pH = 6.1) at 37°C for 6 h. Staining solution was removed and coverslips were overlaid with 70% glycerol and then images were captured under a phase contrast microscope. The number of cells with β-galactosidase staining was calculated in proportion to unstained cells.

### Statistical analysis

All data is presented as mean ± standard error of the mean (SEM) of 3–5 parallel GFBL and SFBL lines from repeated experiments. Statistical analysis was done by using Student's t-test and multiple comparisons were performed by ANOVA, p<0.05 was considered statistically significant. Values obtained from the RT-PCR by the comparative Ct-method were Log2 transformed for statistical testing.

## Results

### Characterization of cell morphology, proliferation and total protein production in GFBLs and SFBLs in 3D cultures

**Figure 1:**
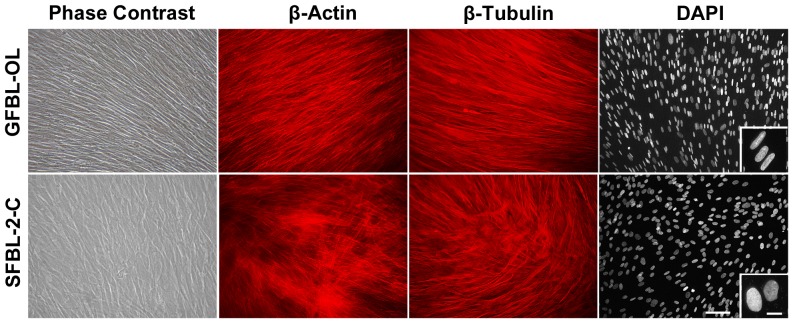
Morphological characterization of GFBLs and SFBLs in 3D cultures. Representative images of GFBL and breast SFBL 3D cultures seven days post-seeding as assessed by phase contrast microscopy, β-actin and β-tubulin immunostaining and DAPI nuclear staining. GFBLs adopted a more elongated shape of the cell body and nucleus, whereas SFBLs were wider in morphology. Cells In GFBL cultures were arranged parallel to each another, while they were more randomly distributed in SFBL cultures. Magnification bars: 50 µm (main figure); 10 µm (insert).

**Figure 2:**
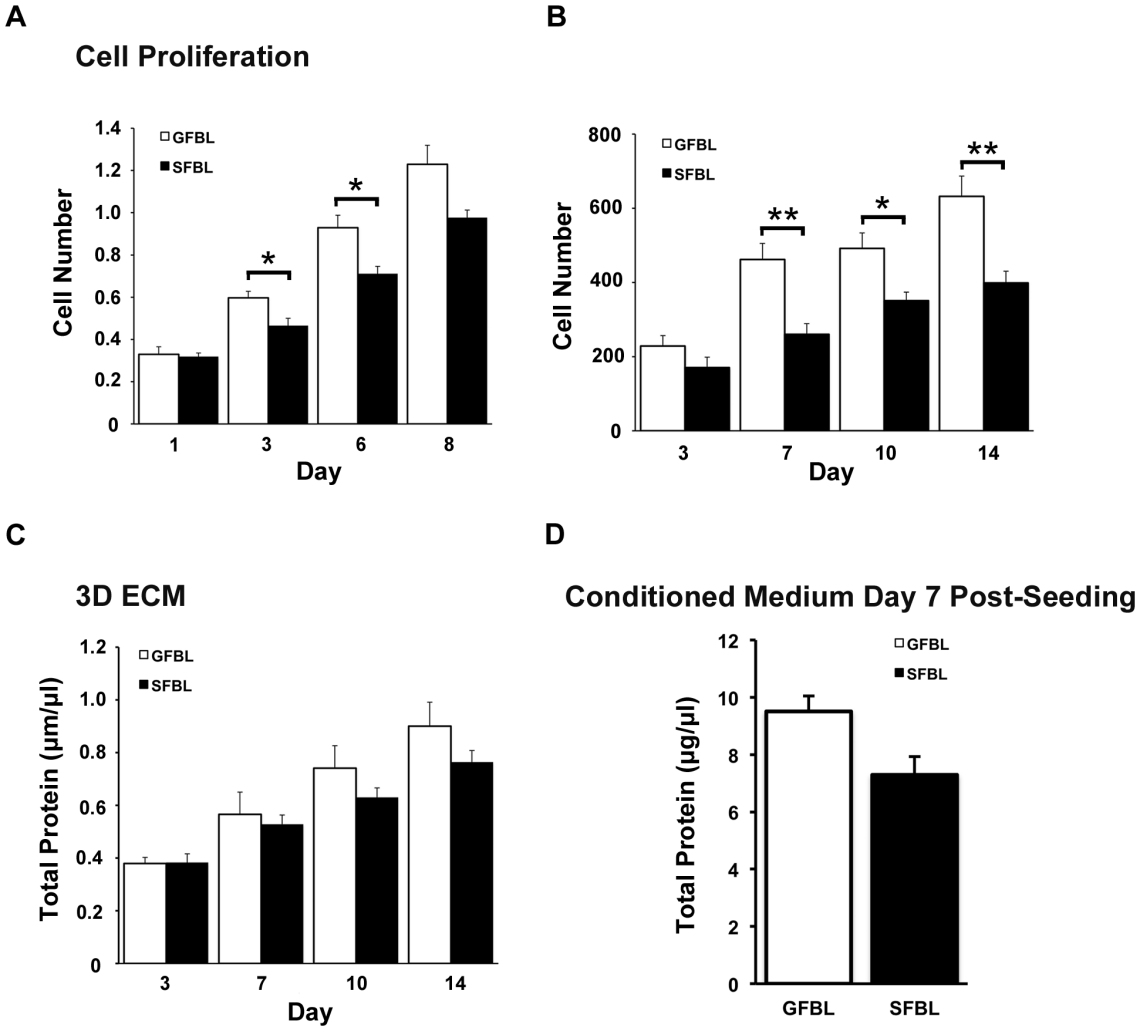
GFBLs proliferate faster but produce similar amount of total proteins as SFBLs. Cell proliferation was assessed at both low (A) and high (B) starting cell density, using the MMT assay and measuring total RNA concentration, respectively. Quantification of total proteins produced into 3D ECM (C) and conditioned medium (D). Results are representative of three repeated experiments. Results show mean +/− SEM from five (A, C, D) and three (B) parallel GFBL and breast SFBL lines from different donors (*p<0.05; Student's t-test).

In order to create 3D cultures, fibroblasts were seeded at high density on cell culture-treated plastic culture dishes reaching confluence within 24 h after seeding. Over time, cells proliferated and deposited ECM becoming embedded in their own matrix. At seven and 21 days post-seeding, the cultures were composed of about 3–5 and 7–10 cell layers, respectively (data not shown). To compare cell morphology and organization in the cultures, we used phase contrast microscopy, immunostaining and nuclear staining at seven days post-seeding (Figure 1). GFBLs adopted a more spindle-like and elongated cell shape of the cell body and nucleus, whereas SFBLs were larger and wider in morphology (Figure 1). In addition, in GFBL cultures, cells were arranged parallel to one another, while in SFBL cultures they were more randomly distributed. Similar cell morphology and organization was apparent also at day 14 and 21 after seeding (data not shown). Next, we compared cell growth in cultures that were seeded either at low (subconfluent) or high (confluent) density. At low density, GFBLs proliferated significantly faster than SFBL at three and six days after seeding (Figure 2A). At day eight when cultures had reached confluence, cell number in GFBL cultures was also greater than in SFBL cultures, but the difference was not statistically significant. Likewise, at high-density conditions (Figure 2B), there was a significantly greater number of GFBLs than SFBLs by day seven, and this difference remained statistically significant at day 10 and 14 post-seeding. The abundance of total protein in the GFBL 3D ECM fraction was also higher at 3, 7, 10 and 14 days after cell seeding (Figure 2C), but the difference did not reach statistically significance. In addition, at seven days post-seeding, the concentration of total proteins secreted into the CM by GFBLs and SFBLs was not significantly different (Figure 2D).

### GFBLs and SFBLs display distinct gene expression profiles in 3D cultures

**Figure 3:**
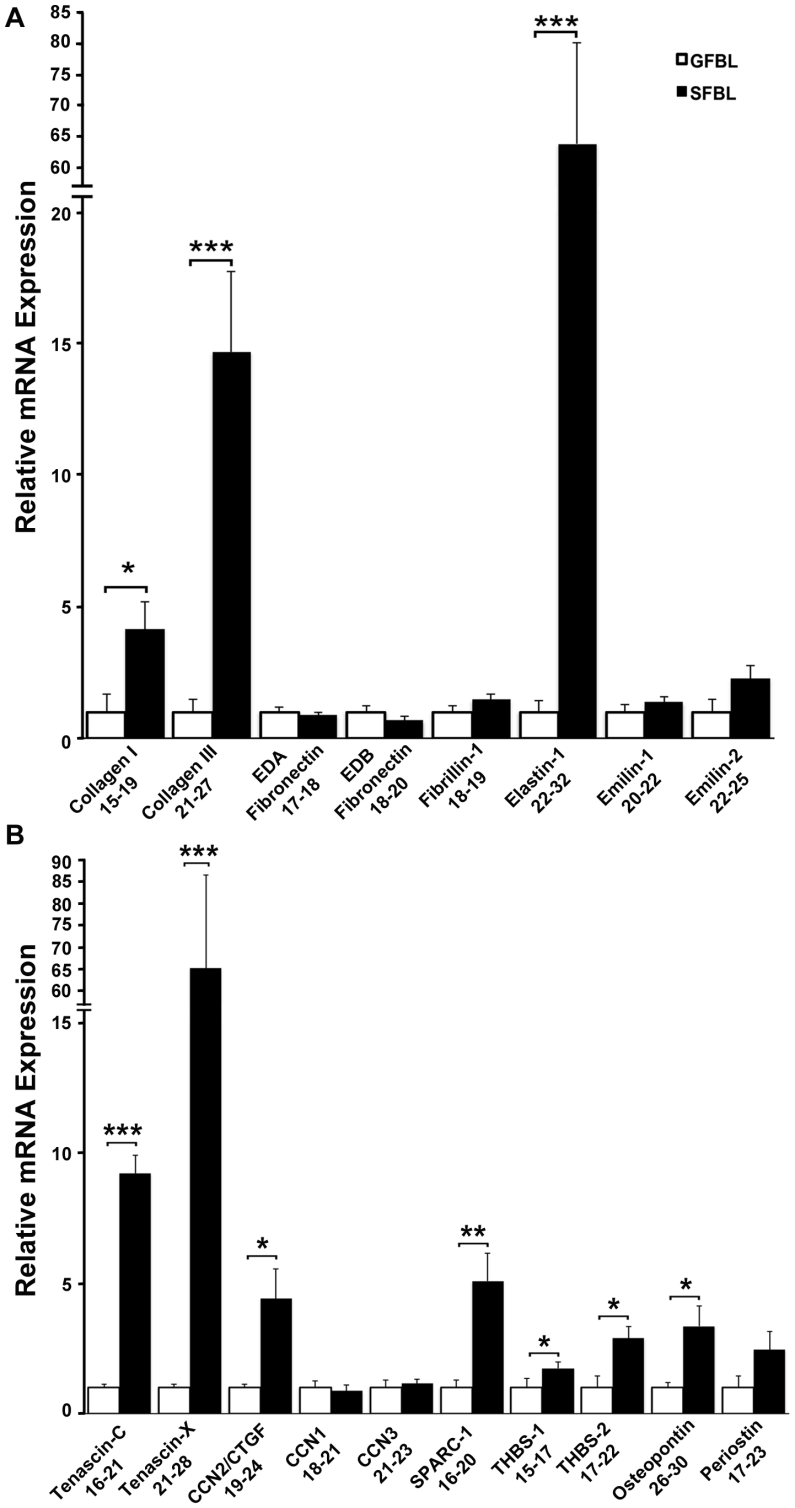
SFBLs express significantly higher levels of genes for ECM molecules. Relative expression of mRNA for fibrillar ECM proteins (A) and matricellular proteins (B) in GFBL and breast SFBL 3D cultures seven days post-seeding. Expression of mRNA was calculated relative to GFBLs. (A) SFBLs express significantly greater levels of type I and III collagen, and elastin. (B) SFBLs express significantly greater amounts of tenascin-C and –X, CCN2/CTGF, SPARC-1, thrombospondin-2 (THBS-2) and osteopontin. Results show mean +/− SEM from five parallel cell lines from different donors (*p<0.05, **p<0.01, ***p<0.001; Student's t-test). Range of Ct-values obtained from real-time RT-PCR is indicated below each gene name.

The hallmark of scar formation is excess accumulation of ECM due to increased ECM deposition and reduced ECM turnover [2]. Therefore, we first compared expression of ECM protein genes between GFBLs and SFBLs in the 3D cultures seven days post-seeding. Fibrillar ECM proteins collagen type I (4.1-fold), collagen type III (14.7-fold) and elastin (64-fold) were significantly elevated in SFBLs (Figure 3A). Apart from elastin, other components of elastic fibers, fibrillin-1, emilin-1 and -2 did not show a significant difference. Emilin-3 expression was negligible in both GFBLs and SFBLs (Ct = 31–34). Furthermore, cellular extra-domain A (EDA)- and EDB-fibronectin isoforms, components of wound ECM, showed no difference between SFBL and GFBL (Figure 3A).

Matricellular proteins were also more highly expressed by SFBLs (Figure 3B). SFBLs showed significantly greater expression of tenascin-C (9.6-fold), tenascin-X (63.3-fold), SPARC-1 (5.0-fold), osteopontin (3.3-fold), thrombospondin-1 (1.7-fold) and thrombospondin-2 (2.9-fold). Out of the three CCN family members, only CCN2/CTGF was significantly higher expressed in SFBLs (4.4-fold) (Figure 3B). Hevin-1 and -2 expression was negligible in both GFBLs and SFBLs (Ct = 30-32; data not shown).

**Figure 4:**
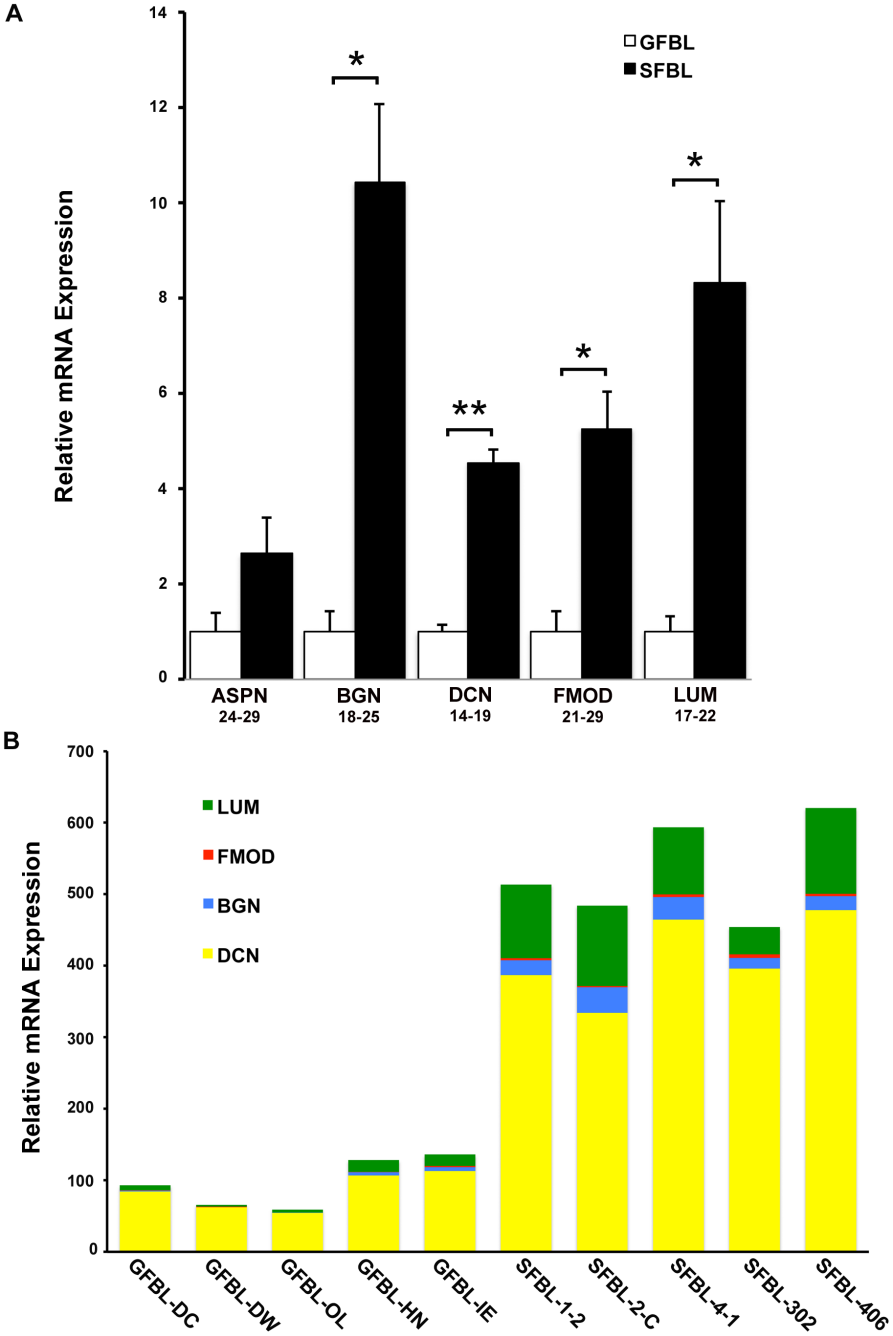
SFBLs express significantly higher levels of small leucine-rich proteoglycans. Relative expression of mRNA for small leucine-rich proteoglycans in GFBL and breast SFBL 3D cultures seven days post-seeding. (A) Expression of mRNA was calculated relative to GFBLs. Results show mean +/− SEM from five parallel cell lines from different donors (*p<0.05, **p<0.01, ***p<0.001; Student's t-test). Range of Ct-values obtained from real-time RT-PCR is indicated below each gene name. (B) Results show relative proportions of mRNA for significantly differently expressed SLRPs in five GFBL and breast SFBL lines from different donors. Decorin was most highly expressed, while fibromodulin was the least abundant in both GFBLs and SFBLs. Expression of total SLRP mRNA was up to 10-fold higher in SFBLs compared to GFBLs. Results were generated by the 2^(Ctreference-Cttarget)^ method. ASPN: Asporin; BGN: Biglycan; DCN: Decorin; FMOD: Fibromodulin; LUM: Lumican.

Among the five small leucine-rich proteoglycans (SLRPs) analyzed, SFBLs showed significantly greater expression for biglycan (10.2-fold), decorin (4.4-fold), fibromodulin (5.3-fold) and lumican (8.4-fold), while expression of asporin showed no difference (Figure 4A). Expression of the four differently expressed SLRPs was also assessed in proportion to one another, using the 2^(Ctreference-Cttarget)^ method (Figure 4B). Decorin was most highly expressed, while fibromodulin was the least abundant in both GFBLs and SFBLs. Expression of total SLRP mRNA was up to 10-fold higher in SFBLs compared to GFBLs (Figure 4B).

**Figure 5:**
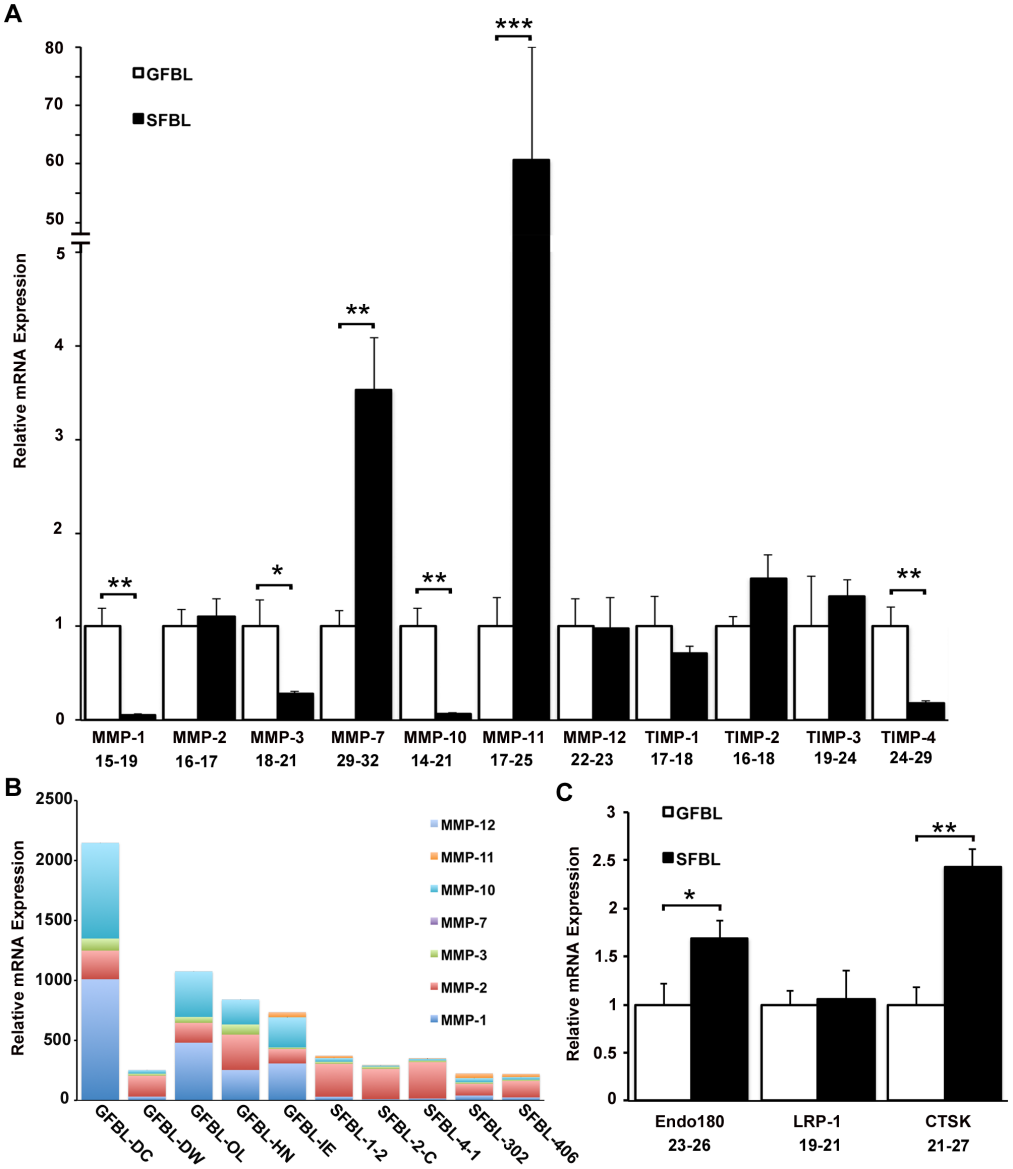
Expression of MMPs and genes for intracellular ECM turnover is distinct in GFBLs and SFBLs. Expression of mRNA was analyzed in 3D cultures seven days post-seeding. (A) GFBLs express significantly higher levels of MMP-1, MMP-3, MMP-10 and TIMP-4, and lower levels of MMP-7 and MMP-11 than SFBLs. Expression of mRNA was calculated relative to GFBLs. Results show mean +/− SEM from five parallel cell lines from different donors (*p<0.05, **p<0.01, ***p<0.001; Student's t-test). (B) Results show relative proportions of individual MMPs expressed by the five parallel GFBL and breast SFBL lines. Total MMP expression was up to ten-fold greater in GFBLs compared to SFBLs. In GFBLs, MMP-1, MMP-2 and MMP-10 were the most abundant MMPs, while SFBLs expressed predominantly MMP-2. In general, MMP-7 and MMP-11 were expressed at very low levels compared to other MMPs. Results were generated by the 2^(Ctreference-Cttarget)^ method. (C) SFBLs express significantly higher levels of Endo180 and CTSK than GFBLs. Endo180: also known as CD280; CTSK: Cathepsin K; LRP-1: Low density lipoprotein receptor-related protein-1. A and C: Range of Ct-values obtained from real-time RT-PCR is indicated below each gene name.

Having established that SFBLs expressed higher levels of many ECM protein genes, we assessed whether molecules involved in ECM turnover, namely MMPs and molecules that mediate internalization and intracellular degradation of ECM, are differently expressed in GFBLs and SFBLs. MMP expression was in general markedly greater in GFBLs than in SFBLs (Figure 5A and B). GFBLs expressed significantly more MMP-1 (18.1-fold), MMP-3 (3.6-fold) and MMP-10 (16.1-fold), while SFBLs expressed significantly more MMP-7 (3.5-fold) and MMP-11 (60.6-fold) (Figure 5A). MMP-13 expression was negligible in both GFBLs and SFBLs (Ct = 32–34). Analysis of overall proportions of MMPs by the 2^(Ctreference-Cttarget)^ method showed that the total MMP expression was up to ten-fold greater in GFBLs compared to SFBLs (Figure 5B). In GFBLs, MMP-1, MMP-2 and MMP-10 were the most abundant MMPs, while SFBLs expressed predominantly MMP-2. In general, MMP-7 and MMP-11 were expressed at very low levels compared to other MMPs (Figure 5B). Among the four members of the TIMP family (TIMP1-4), only TIMP-4 was more highly expressed in GFBLs (5.7-fold), while the other genes showed no significant difference (Figure 5A). Genes involved in intracellular turnover of ECM, the collagen endocytosis receptor Endo180 (CD280; 1.7-fold) and lysosomal enzyme cathepsin K (CTSK; 2.4-fold), were significantly more expressed in SFBLs compared to GFBLs, while there was no difference in the expression of low density lipoprotein receptor-related protein-1 (LRP-1), which is involved in ECM endocytosis (Figure 5C).

**Figure 6:**
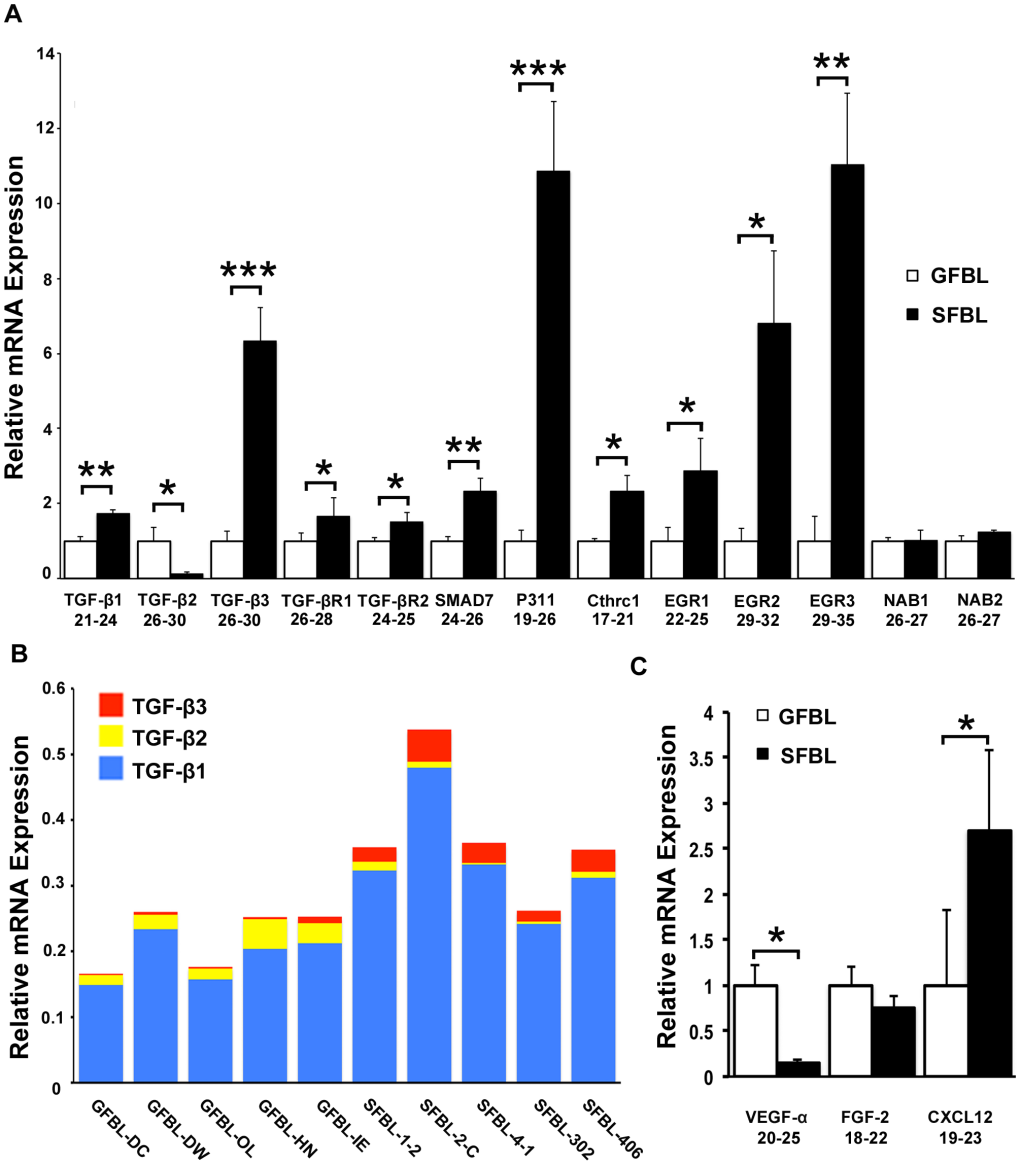
Distinct expression of TGF-β signaling and pro-angiogenic molecules in SFBLs and GFBLs. Expression of mRNA was analyzed in 3D cultures seven days post-seeding. (A) Expression of TGF-β signaling related molecules in SFBLs relative to GFBLs. SFBLs expressed significantly elevated levels of TGF-β1 and -β3, SMAD7, P311, Cthrc1, EGR2 and EGR3, while GFBLs expressed significantly higher amount of TGF-β2. (B) Relative proportion of TGF-β1, -β2 and -β3 in individual GFBL and breast SFBL lines from different donors assessed by the 2^(Ctreference-Cttarget)^ method. TGF-β1 was the major isoform expressed by all cell lines, and SFBL lines in general showed a higher total TGF-β expression compared to GFBLs. Relative proportions of TGF-β2 and TGF-β3 were higher in GFBLs and SFBLs, respectively. (C) Expression of pro-angiogenic factors. GFBLs showed significantly higher expression of VEGF-α than SFBLs. A and C: mRNA levels were calculated relative to GFBLs, and results show mean +/− SEM from five parallel cell lines from different donors (*p<0.05, **p<0.01, ***p<0.001; Student's t-test). Range of Ct-values obtained from real-time RT-PCR is indicated below each gene name. TGF-βR1: TGF-β Receptor 1; TGF-βR2: TGF-β Receptor 2; CXCL12: also known as SDF-1α (Stromal-Derived Factor-1α); Cthrc1: Collagen triple helix repeat containing 1; EGR: Early Growth Response; NAB: NGFI-A Binding Protein.

TGF-β signaling pathway is overactive in fibrosis and scar formation and promotes ECM deposition while down regulating matrix turnover [3]. TGF-β1 (1.7-fold) and TGF-β3 (6.4-fold) were significantly more highly expressed in SFBLs, while TGF-β2 (7.0-fold) was higher in GFBLs (Figure 6A). When the proportional expression of TGF-β1, -β2 and -β3 was assessed in individual cell lines by the 2^(Ctreference-Cttarget)^ method, TGF-β1 was the major isoform expressed by all cell lines, and SFBL lines in general showed a higher total TGF-β expression compared to GFBLs (Figure 6B). SFBLs expressed on average a higher relative proportion of TGF-β3 (about 8%) compared to GFBLs (about 2%) out of the total TGF-β mRNA, while the respective proportions for TGF-β2 for GFBLs and SFBLs were about 10% and 2% (Figure 6B). The intracellular protein P311, that promotes steady-state TGF-β1, -β2 and -β3 translation [30], showed a significantly greater expression (10.5-fold) in SFBLs (Figure 6A). TGF-β receptors TGF-βR1 (1.7-fold) and TGF-βR2 (1.5-fold) were also significantly more highly expressed by SFBLs. The Early Growth Response 1 (EGR1), EGR2 and EGR3, transcription factors that mediate TGF-β-induced signaling, and are highly expressed in fibrosis [31]–[33], were also significantly elevated (2.9-, 6.8- and 10.2-fold, respectively) in SFBLs, while expression of transcriptional EGR repressors, NGFI-A binding protein (NAB) 1 and NAB2 [34], showed no differences (Figure 6A). SFBLs also expressed significantly higher levels of SMAD7 (2.3-fold), an inhibitor of SMAD2/3 signaling [35], and Collagen triple helix repeat containing-1 (Cthrc1; 2.3-fold) that can suppress TGF-β signaling [36], [37].

Pro-angiogenic factors, including VEGF-α, CXCL12 (SDF-1α) and FGF-2, are important regulators of angiogenesis during wound healing, while abnormal vascularization has been linked with scar formation and fibrosis [38], [39]. The findings showed that expression of VEGF-α was significantly greater (7.0-fold) in GFBLs. In contrast, CXCL12 (SDF-1α) was 2.7-fold elevated in SFBLs. FGF-2 showed no difference between GFBLs and SFBLs (Figure 6C).

**Figure 7:**
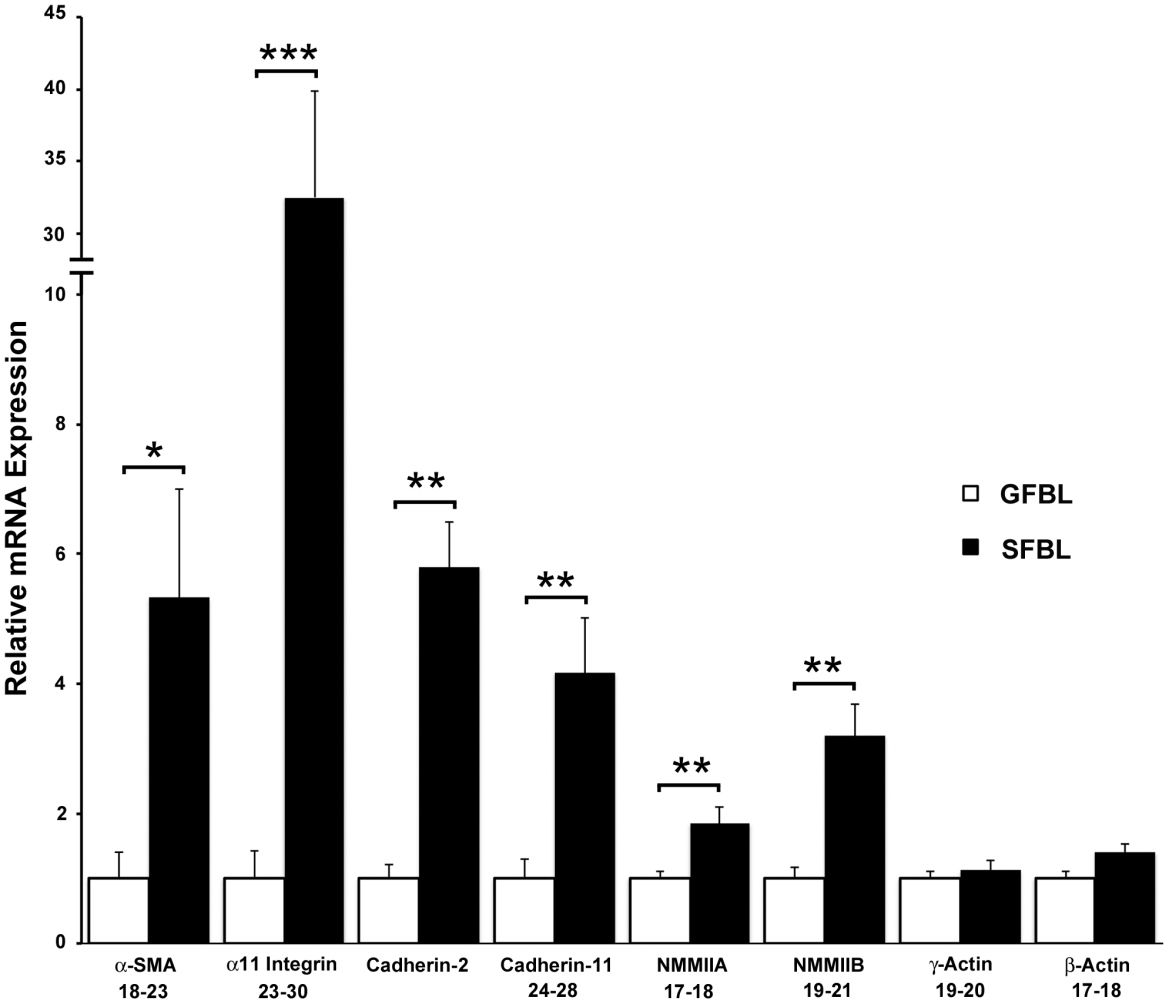
Greater expression of contractility and myofibroblast-associated genes in SFBLs. Expression of mRNA was analyzed in 3D cultures seven days post-seeding, and results are presented relative to GFBLs. Results show mean +/− SEM from five parallel GFBL and breast SFBL lines from different donors (*p<0.05, ** p<0.01, ***p<0.001; Student's t-test). Range of Ct-values obtained from real-time RT-PCR is indicated below each gene name. α-SMA: α-Smooth Muscle Actin; NMMIIA: Non-Muscle Myosin IIA; NMMIIB: Non-Muscle Myosin IIB.

Myofibroblast phenotype and their increased contractility has been linked to a profibrotic wound healing response and scar formation [7]. Analysis of genes in this category showed that α-SMA (5.3-fold), Non-Muscle Myosin IIA (NMMIIA; 1.9-fold) and NMMIIB (3.2-fold) involved in cell contraction [40], and α11 integrin (32.3-fold) that regulates myofibroblast differentiation [41], were all significantly more highly expressed in SFBLs compared to GFBLs (Figure 7). In addition, cell-cell adhesion molecules cadherin-2 and myofibroblast-associated cadherin-11, involved in cell-mediated ECM contraction [42], were significantly higher (5.8- and 4.2-fold, respectively) in SFBLs than GFBLs (Figure 7). Relative mRNA expression data for individual breast skin and gingival cell lines for all the genes that exhibited Ct-values<30 in either cell type are shown in Table S3.

### Gene expression between GFBL and SFBL is significantly different in 3D culture over time

**Figure 8:**
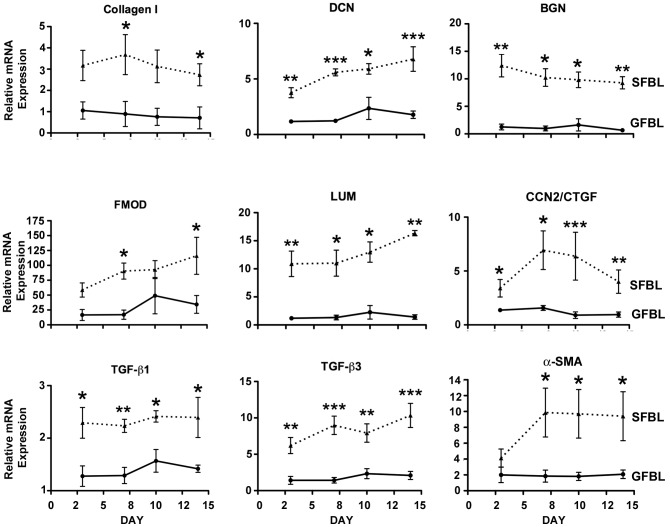
Tissue-specific gene expression is stable over time in 3D cultures. Results show mean +/− SEM of relative mRNA expression from five parallel GFBL and breast SFBL lines from different donors, and were calculated relative to one GFBL line. Statistically comparison performed between GFBLs and SFBLs at each time point are shown (*p<0.05, **p<0.01, ***p<0.001; Student's t-test and two-way ANOVA). DCN: Decorin; BGN: Biglycan; FMOD: Fibromodulin; LUM: Lumican; CCN2/CTGF: Connective Tissue Growth Factor; α-SMA: α-Smooth Muscle Actin.

To determine whether the noted differences in gene expression between GFBLs and SFBLs depended on culture time, a set of genes was analyzed at days 3, 7, 10 and 14 post-seeding. The analyzed genes showed consistent and significantly different level of expression between GFBL and SFBL over the entire experimental period (Figure 8). No statistically significant differences in gene expression over time from days 3 to 14 in SFBLs or GFBLs were found.

### Comparison of gene expression between GFBLs and SFBLs from breast and abdominal skin

To test whether differences between GFBLs and breast SFBLs used in the above experiments depended on the origin of skin cells, we compared expression of a set of genes in three independent SFBL lines from abdominal skin to the above cell lines. Out of the 28 genes analyzed, 27 genes showed similar expression pattern in both breast and abdominal SFBLs relative to GFBLs (Figure S1). In addition, expression of TGF-β1, DCN and BGN was even more elevated in the abdominal (on average 6.0-, 26.8- and 69.5-fold higher, respectively) than in breast (1.8-, 4.4- and 10.2-fold higher, respectively) SFBLs compared to GFBLs. Likewise, expression of CXCL12 (SDF-1α) by breast and abdominal SFBLs was on average 2.7- and 25.2-fold higher than in GFBLs, respectively. MMP-2 was the only gene in this comparison that was expressed at a markedly higher level by abdominal (on average 3.5-fold), but not by breast SFBLs, relative to GFBLs (Figure S1).

### Characterization of protein abundance in the conditioned medium

**Figure 9:**
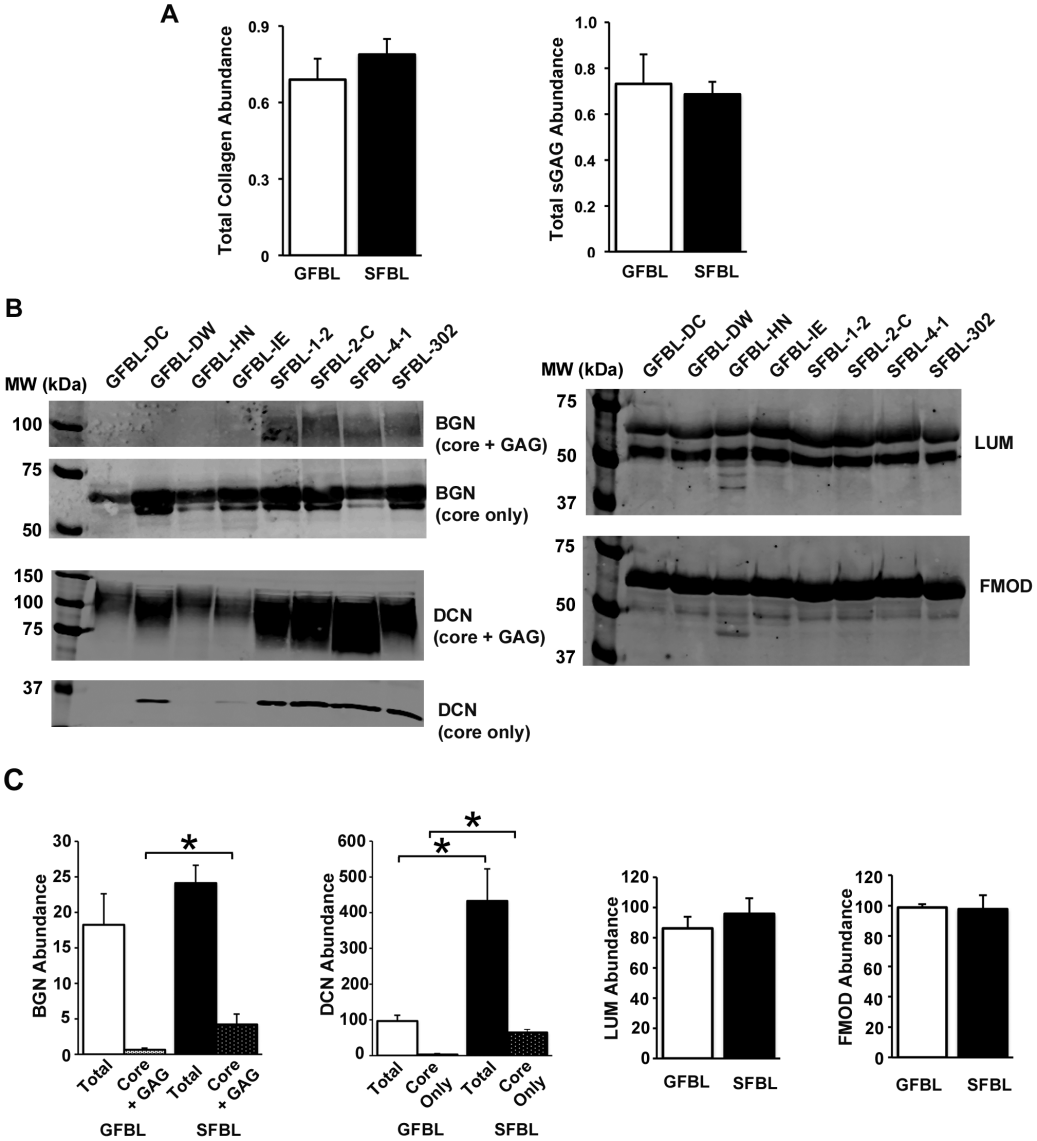
Abundance of collagen, glycosaminoglycans and SLRPs in 3D cultures. (A) Quantification of total collagen and sulphated glycosaminoglycans (sGAG) secreted into the conditioned medium (CM). Western blotting (B) and quantification (C) of small leucine-rich proteoglycans (SLRPs) biglycan (BGN), decorin (DCN), lumican (LUM) and fibromodulin (FMOD) in the CM. Cells secreted both the proteoglycan form (core protein with attached GAG chains; core + GAG) and core-proteins devoid of GAG chains (core only) of SLRPs. SFBLs secreted significantly elevated levels of BGN proteoglycan (core + GAG) and DCN compared to GFBLs. Identity of the proteoglycan and core protein fractions was confirmed in a set of samples by enzymatic digestion with chondroitinase ABC and keratanase prior to electrophoresis and Western blotting (data not shown). For better visualization, contrast of the image for the immunoblot for BGN proteoglycan fraction (core + GAG) shown is increased relative to image showing core protein only (B). Quantitative results shown in (C) were generated with corresponding non-enhanced image. For BGN and DCN, the results show total proteoglycan pool and core protein fractions compared separately. Total: Results for proteoglycan (core + GAG) and (core only) are combined to represent the total proteoglycan pool; Core only: Quantification of core protein fraction only. Results show mean +/− SEM from four to five parallel GFBL and breast SFBL lines from different donors (*p<0.05; Student's t-test).

Having established that GFBLs and SFBLs have significantly different gene expression profile, we assessed whether these differences were also present when a set of proteins produced by GFBLs and breast FBLs in the CM were analyzed. Results showed no significant differences in the secretion of total collagen or sulphated GAGs in CM between SFBLs and GFBLs (Figure 9A). However, Western blotting demonstrated that SFBLs secreted significantly greater amount of total decorin proteoglycan (4.5-fold) and core protein (12.1-fold) (Figure 9B and C). In addition, the level of biglycan proteoglycan (core + GAG) was significantly greater (5.3-fold) in SFBLs than GFBLs (Figure 9B and C). There were no differences in total fibromodulin and lumican abundance (Figure 9B and C).

**Figure 10:**
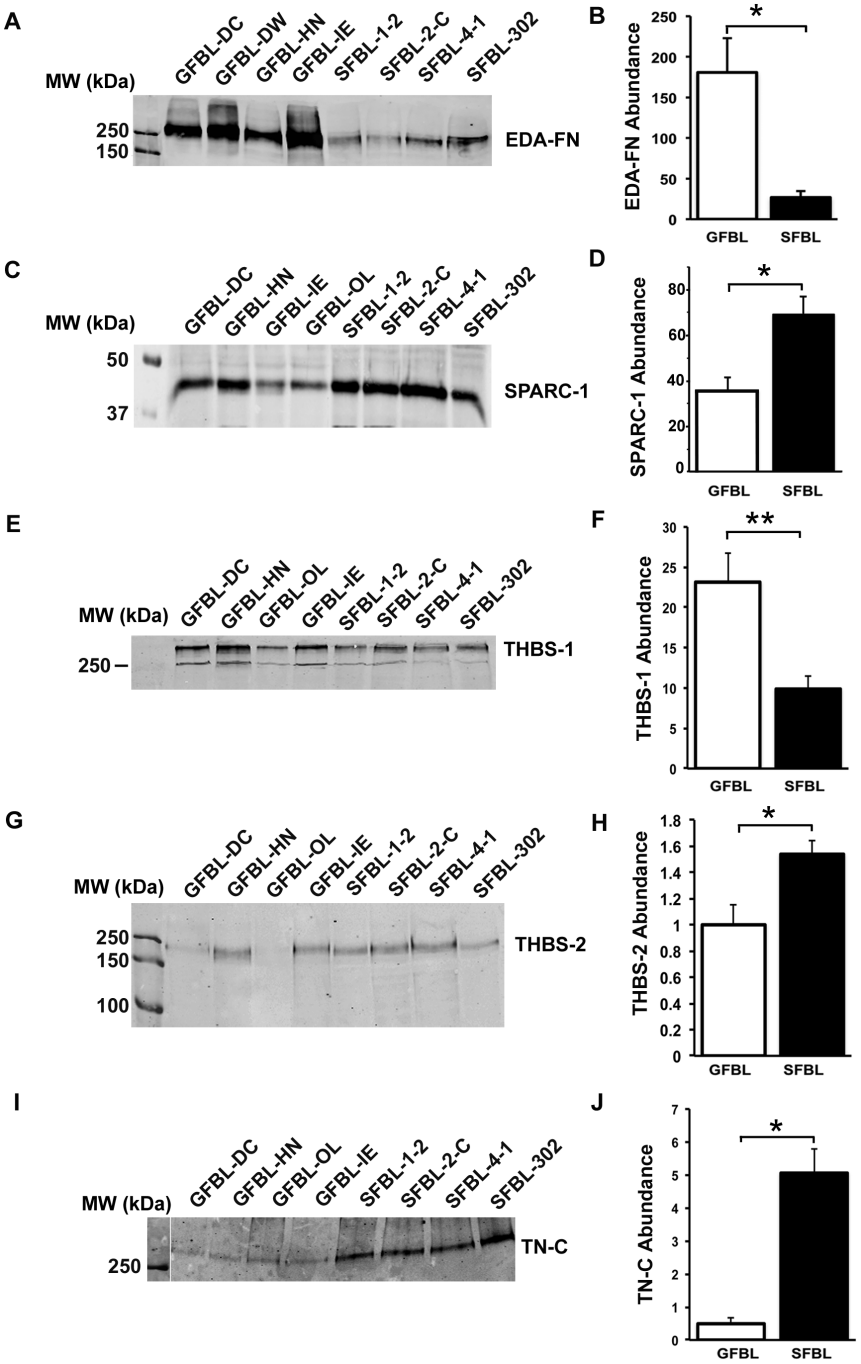
Abundance of EDA-fibronectin, SPARC-1, and thrombospondin-1 and -2 in the conditioned medium of 3D cultures. (A, C and E) Western blotting and quantification (B, D and F) of Extra-domain A (EDA)-fibronectin (EDA-FN) (A and B), SPARC-1 (C and D), thrombospondin-1 (THBS-1) (E and F) and thrombospondin-2 (THBS-2) (G and H) in the conditioned medium of the 3D cultures. GFBLs secreted significantly higher amounts of EDA-FN (A and B) and thrombospondin-1 (E and F), while SFBLs produced significantly elevated amounts of SPARC-1 (C and D) THBS-2 (G and H), and tenascin-C (I and J). Results show mean +/− SEM from four to five parallel GFBL and breast SFBL lines from different donors from two to three parallel experiments (*p<0.05; Student's t-test).

Gene expression analysis showed that SFBLs expressed significantly elevated levels of tenascin-C, SPARC-1, thrombospondin-1 and -2, and osteopontin (Figure 3B). Therefore, we assessed the abundance of corresponding proteins in the CM by Western blotting. We also quantitated the levels of EDA-fibronectin that did not show difference between GFBLs and SFBLs in mRNA expression (Figure 3A). Findings showed that GFBLs secreted significantly elevated amount of EDA-fibronectin (Figure 10A and B) and thrombospondin-1 (Figure 10E ad F), while SFBLs produced significantly higher levels of tenascin-C, SPARC-1 (Figure 10C and D) and thrombospondin-2 (Figure 10G-H). Osteopontin, that showed low expression at mRNA level (Ct = 26–30), was undetectable by Western blotting.

**Figure 11:**
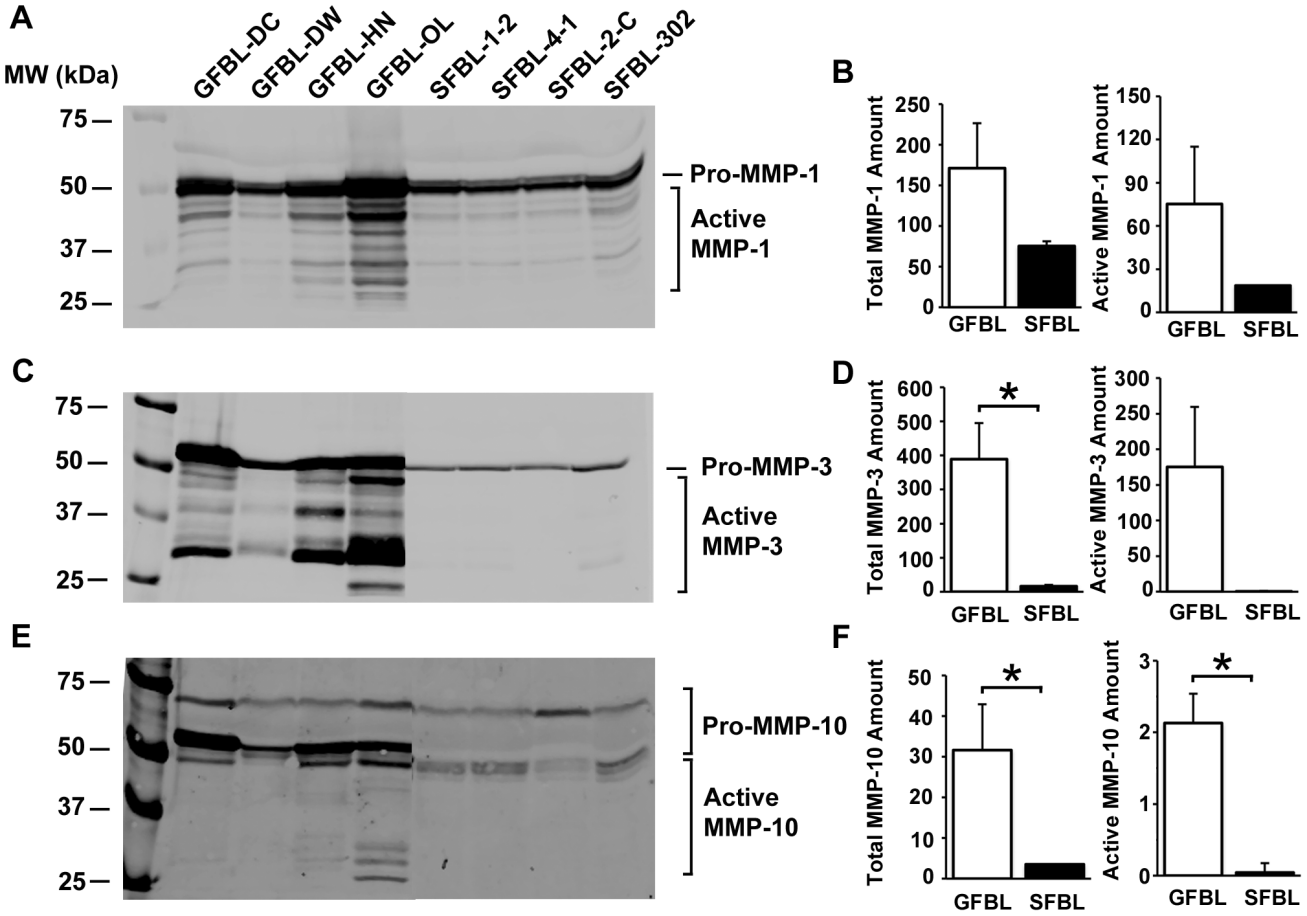
GFBLs secrete significantly greater amount of MMPs compared to SFBLs. Western blotting of MMP-1 (A), MMP-3 (C) and MMP-10 (E), and quantification of corresponding MMPs (B, D and F, respectively) in the conditioned medium. Results show mean +/− SEM from four to five parallel GFBL and breast SFBL lines from different donors (*p<0.05; Student's t-test). D: Statistical testing of active MMP-3 levels between GFBLs and SFBLs was not possible due to low level of detectable active protein in SFBLs. Active and preforms of the enzymes were detected by pretreatment of a set of samples with or without APMA prior to gelelectrophoresis and Western blotting (data not shown).

Gene expression data had shown that GFBLs expressed significantly greater levels of MMP-1, MMP-3 and MMP-10. Analysis of the CM showed that GFBLs also produced significantly elevated amount of total and active MMP-3 (16.3- and more than 300-fold, respectively) and MMP-10 (9.0- and 50.7-fold, respectively) as compared to SFBLs (Figure 11C-F). The level of total and active MMP-1 was also higher (2.3- and 4.0-fold, respectively) in GFBL cultures, but the difference to SFBLs did not reach statistical significance (Figure 11A and B).

**Figure 12:**
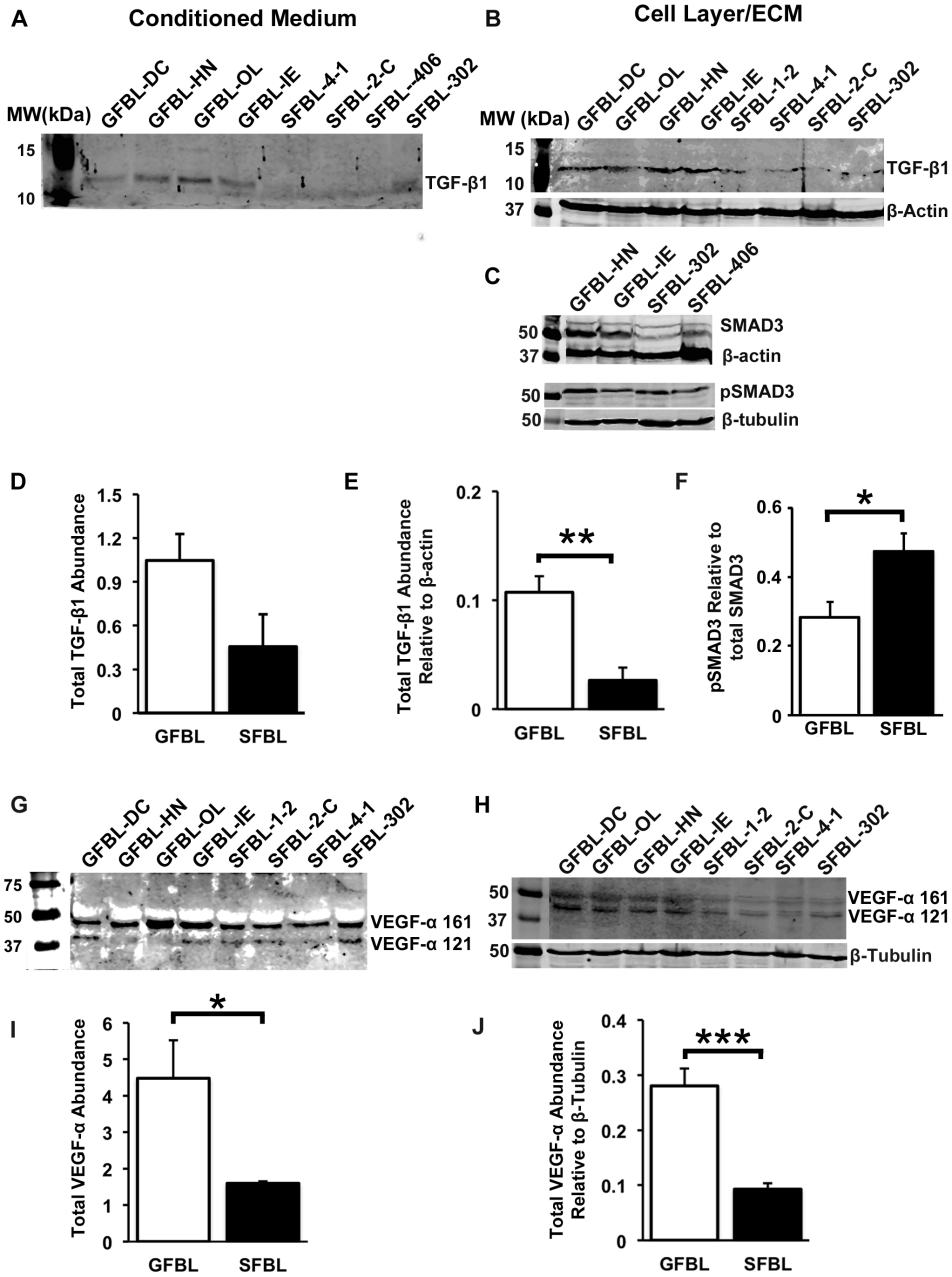
Quantification of TGF-β1 and VEGF-α in GFBL and SFBL 3D cultures. Western blotting and quantification of TGF-β1 in the cell/ECM fraction (A and D, respectively) and in the conditioned medium (B and E, respectively). GFBLs produced significantly elevated amounts of TGF-β1 in the cell/ECM layer compared to SFBLs. Western blotting (C) and quantification (F) of total SMAD3 and phosphorylated SMAD3 (pSMAD3). Level of pSMAD3 relative to total SMAD3 was significantly elevated in SFBLs compared to GFBLs. Western blotting and quantification of VEGF-α in the cell/ECM fraction (G and I, respectively), and in the conditioned medium (H and J, respectively). GFBLs produced significantly elevated levels of VEGF-α compared to SFBLs. G and H: Both cell types produced two distinct VEGF-α isoforms (121 and 161 amino acid splice variants). Results show mean +/− SEM from four to five parallel GFBL and breast SFBL lines from different donors (*p<0.05, **p<0.01, ***p<0.001; Student's t-test). Sample loading was normalized for β-actin or β-tubulin levels. The results represent two repeated experiments.

Gene expression analysis showed differential expression of many TGF-β signaling associated genes in GFBLs and SFBLs. Therefore, total TGF-β1 (latent and active) abundance was analyzed by Western blotting in the CM. Latent-TGF-β1 can also avidly bind to the ECM [3]. Thus, we assessed its abundance also in the cell layer/ECM fraction. In contrast to mRNA expression data, there were markedly increased levels of TGF-β1 in the GFBL cell layer/ECM (3.8-fold) (Figure 12A and D) and CM (2.3-fold) (Figure 12B and E) than in SFBL cultures. To elucidate the activation of TGF-β signaling pathway, we investigated the relative levels of phosphorylated SMAD3 (pSMAD3). Interestingly, there was a significantly greater steady-state abundance of pSMAD3 relative to total SMAD3 (1.7-fold) in SFBLs compared to GFBLs (Figure 12C and F). TGF-β signaling can collaborate with other pro-fibrotic pathways, including ERK1/2 and p38 of the MAPK pathway, and β-catenin pathway [43], [44]. However, no differences between GFBLs and SFBLs were found when the levels of corresponding phosphorylated proteins were compared to respective total proteins by Western blotting (data not shown).

RT-PCR data indicated that GFBLs expressed strongly elevated levels of pro-angiogenic VEGF-α. This growth factor can also become sequestered in the ECM [38]. Results showed significantly higher level of VEGF-α in both GFBL CM (2.7-fold higher) and cell/ECM layer (3.0-fold higher) than in SFBL cultures (Figure 12G-J).

**Figure 13:**
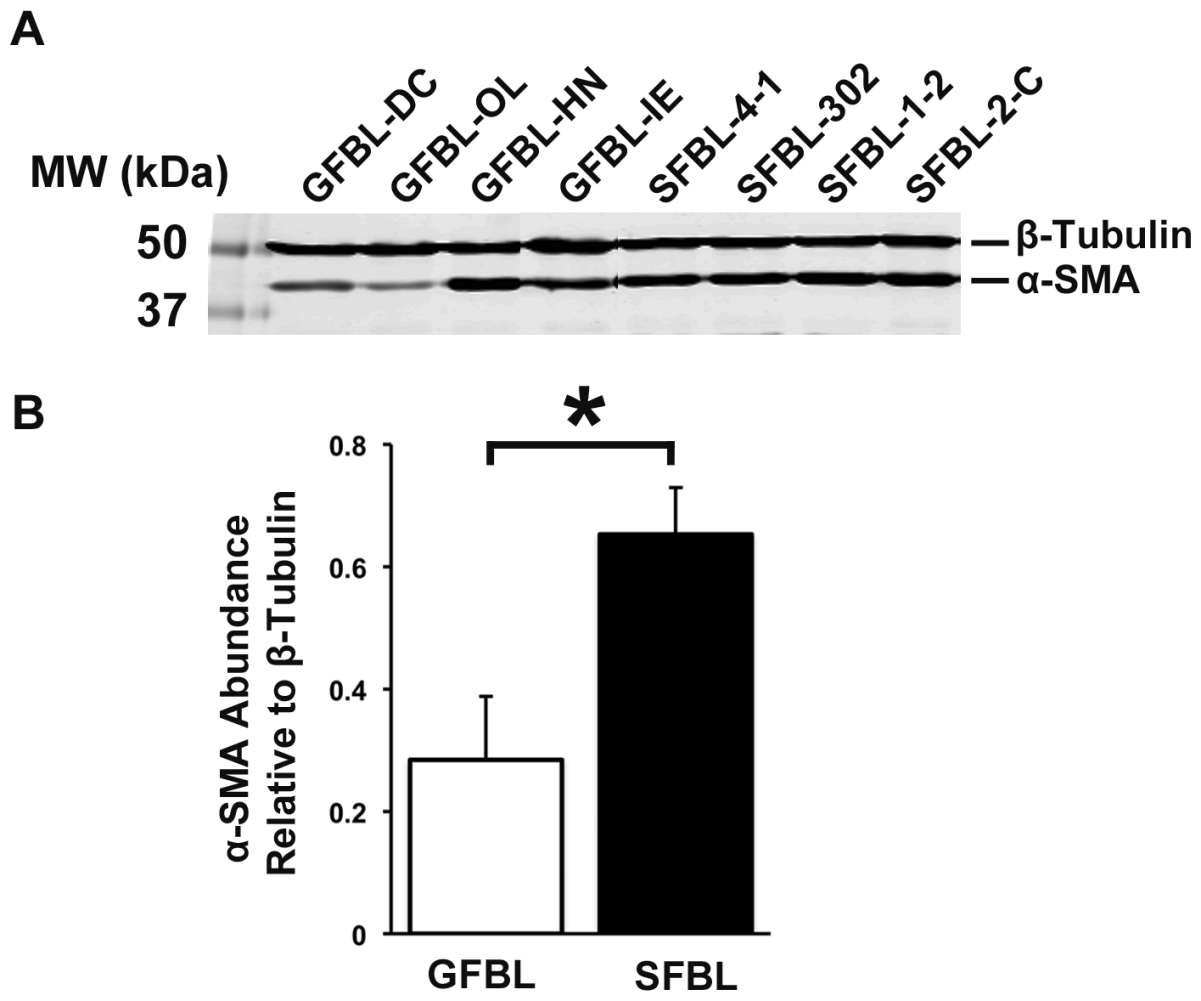
Significantly greater amount of α-Smooth Muscle Actin in SFBLs. Results show Western blotting (A) and quantification (B) of α-Smooth Muscle Actin (α-SMA; mean +/− SEM) from four to five parallel GFBL and breast SFBL lines from different donors (*p<0.05; Student's t-test). Sample loading was normalized for β-tubulin levels. Analysis was performed in 3D cultures seven days post-seeding.

SFBL expressed significantly higher levels of myofibroblast-associated genes, including α-SMA, than GFBLs suggesting that the SFBL cultures may have contained elevated numbers of myofibroblasts. To test this, we first analyzed α-SMA levels by Western blotting. Findings confirmed significantly elevated abundance (2.3-fold) of α-SMA in SFBLs (Figure 13). To assess whether this associated with myofibroblast phenotype, number of cells showing cytoskeletal α-SMA-rich stress fibers were quantified by immunostaining. Findings showed that only about 1% and 4% of GFBL and SFBL were myofibroblasts, respectively. These proportions remained unaltered when the cells were assessed at an early (P5–7) and late (P8–11) passage (data not shown). To find out whether cultures contained any senescent cells that are phenotypically distinct from actively dividing cells [45], we quantified cells positive for senescence-associated β-galactosidase (not shown). At early (P5–7) and late (P8–11) passage, only about 1% and 2.2% of GFBLs and 0.5% and 0.3% of SFBLs showed positive staining for the senescence marker, respectively.

## Discussion

Accumulating evidence from human and animal models have indicated that oral mucosal wound healing is in general significantly faster and associates with a shorter and milder inflammatory response than skin wounds. In addition, wound healing in oral mucosal gingiva results to less scar formation than in similar skin wounds when assessed using clinical, histological and molecular parameters [5], [10], [11], [46]. In the present study, we hypothesized that fibroblast phenotype in oral mucosal gingiva and skin is distinct which may in part contribute to the different wound healing responses. To this end, we utilized a 3D culture model that better mimics the natural ECM niche found *in vivo* than the traditional 2D cultures [18], [19]. In addition, cells were cultured in the presence of 10% serum that has been shown to trigger a wound healing-associated transcriptome in fibroblasts [47]. The key finding was that GFBLs and SFBLs are markedly distinct, the former proliferating faster and expressing higher levels of molecules involved in modulation of inflammation and ECM remodeling (MMPs), while SFBLs displayed significantly higher expression of fibrillar (collagens and elastin) and non-fibrillar (SLRPs and matricellular proteins) ECM proteins, and molecules involved in TGF-β signaling, regulation of myofibroblast phenotype and cell contractility. Thus, GFBLs display a phenotype that may promote faster resolution of inflammation and ECM remodeling, which is characteristic to reduced scar formation, while SFBLs have a profibrotic, scar-prone phenotype. The phenotypic differences were stable over time in culture, suggesting that they are an inherent property of the cells. Furthermore, the key differences did not depend on whether skin cells originated from breast or abdominal skin, both prone to scar formation [23], [24], suggesting that the differences to GFBLs are a general characteristic of SFBLs. Whether this phenotypic difference depends on embryonic origin of the cells remains to be shown, as gingival cells mostly originate from the neural crest while abdominal and breast SFBLs are of mesodermal origin [11], [17]. In any case, the above differences cannot be explained by a phenotypic change of cultured cells into myofibroblasts or cellular senescence as only very small fractions of cells showed positive staining for the respective markers, α-SMA-rich stress fibers and senescence-associated β-galactosidase. In general, myofibroblasts have a more pro-fibrotic ECM producing phenotype [7], while senescent cells express highly MMPs and various inflammatory cytokines, and may associate with a reduced fibrotic tissue response [48], [49].

When individual genes expressed by GFBLs and SFBLs were examined, it was noteworthy that skin cells expressed strongly elevated levels of many ECM molecules, including type I and III collagen and elastin, and matricellular proteins tenascin-C, tenascin-X, SPARC-1, thrombospondin-1 and -2 and osteopontin. Some of these genes can be linked with scar formation and slower wound healing in skin. For instance, increased levels of type I collagen and SPARC-1 associate with scar formation [2], [50], while deletion of thrombospondin-2 or osteopontin accelerates wound healing and reduces scaring [51], [52]. In contrast, deletion of thrombospondin-1 delays wound healing, and associates with reduced levels of active TGF-β in the wounds {52]. The role of type III collagen and tenascin-C is more complex as increased levels have been reported in both fetal skin and oral mucosa that heal with minimal scaring, and in established scars in adult skin [5], [53], [54]. High expression of elastin and tenascin-X by SFBLs is likely related to the tissue origin of cells, as elastin is widely abundant in skin, while tenascin-X is highly expressed only in specific skin areas, particularly in breast. Gingiva almost totally lacks elastin, but the expression of tenascin-X in gingiva is not known [5], [54], [55]. Thus, in the 3D model used in the present study, expression of elastin and tenascin-X reflects SFBL and GFBL phenotypes also found *in vivo*.

SFBLs also expressed significantly elevated levels of SLRPs, decorin, biglycan, lumican and fibromodulin, compared to GFBLs, of which decorin and biglycan were also higher in SFBLs at protein level. SLRPs are multifunctional molecules that have important, partially overlapping, regulatory roles in ECM, and control, for instance, collagen fibrillogenesis. They also bind various growth factors, including TGF-β, and in soluble forms, regulate inflammation and cell signaling [56], [57]. Decorin can also inhibit phagocytosis-mediated intracellular collagen degradation by fibroblasts [58], suggesting that its high expression may perturb this pathway of ECM remodeling in skin. Decorin also has certain anti-angiogenic function, while in certain situations in may also be anti-fibrotic [57]. In addition, decorin and biglycan released from cells or ECM induce pro-inflammatory signaling by binding to toll-like receptors 2 and 4 [57]. In general, expression of SLRPs is spatiotemporally regulated during wound healing, and studies have suggested that scar-free and scar-forming wound healing outcome may in part depend on the balance between these molecules [59]–[61]. However, their exact role in wound healing and scar formation is unclear.

Although SFBLs expressed elevated levels of mRNA of many ECM molecules, including type I collagen, SLRPs and matricellular proteins, no significant differences were found in total amounts of proteins, or in collagen or sulphated GAGs, secreted into CM by GFBLs and SFBLs. Similarly, no differences were found when ECM of the 3D cultures was analyzed for total proteins, collagen and GAGs (unpublished data). However, SFBLs secreted into CM higher levels of decorin, biglycan, tenascin-C, SPARC-1 and thrombospondin-2, while GFBLs produced elevated levels of EDA-FN and thrombospondin-1. SFBLs also deposit significantly elevated levels of biglycan, tenascin-C, and SPARC-1 in the 3D ECM (unpublished results). Therefore, it appears that SFBLs are not prone to produce greater amount of ECM proteins in general, but rather express high levels of certain ECM molecules, that may participate in fibrosis *in vivo*. The above noted mRNA expression differences did not completely correlate with the abundance of corresponding proteins in the 3D cultures. Translation of proteins from mRNA can be controlled at various levels and may involve epigenetic and other mechanisms [62], which may be differently regulated in GFBLs and SFBLs.

Overall, GFBLs expressed higher levels of MMPs both at mRNA and protein level compared to SFBLs, while levels of the major TIMP isoforms (TIMP-1-3) were similar. Particularly, the difference in total MMPs depended on higher expression of MMP-1, MMP-3 and MMP-10 by GFBLs. MMPs are important modulators of inflammation as they degrade chemokines, cytokines, growth factors and their inhibitors activating them or rendering them inactive [63], [64]. For instance, MMP-3, highly expressed by GFBLs, has an anti-inflammatory function [65], while MMP-7, elevated in SFBLs, promotes inflammation by stimulating neutrophil recruitment and activation [66]. Thus, the findings suggest that fibroblast-derived MMPs may contribute to the differential inflammatory responses in gingival and skin wound healing. Of note, scar-free fetal skin wound healing also associates with an increased abundance of MMPs relative to TIMPs compared to adult skin [67]. Higher expression of MMP-3 has also been reported previously in buccal oral mucosal fibroblasts relative to skin fibroblasts using a standard 2D culture [68]. With respect to ECM degradation, MMPs highly expressed by GFBLs have the greatest specificity against fibrillar type I and III collagens, while MMP-7 and MMP-11 more abundant in SFBLs, do not degrade these molecules [63]. Therefore, GFBLs appear to have a higher potential than SFBLs to remodel fibrillar ECM by a proteolytical mechanism. However, molecules involved in internalization and intracellular degradation of collagen, Endo180 and cathepsin K, respectively, were higher in SFBLs. Therefore, ECM remodeling capacity of these cells need to be functionally tested in more detail.

As angiogenesis is critical for wound healing, and aberrant angiogenesis associates with scar formation, we also assessed expression of pro-angiogenic genes by the cells [39]. Findings showed that GFBLs expressed and produced strongly elevated levels of VEGF-α, while SFBLs had a higher expression of CXCL12 (SDF-1α). While VEGF-α is one of the most potent cytokines that promote angiogenesis, CXCL12 has also other functions recruiting inflammatory and progenitor cells [38]. Scar forming skin wounds have higher density of blood vessels as compared to gingival wounds [10]. Therefore, it is possible that GFBLs and SFBLs have different impact on wound healing-associated angiogenesis.

Another property associated with scar formation is persistence or accumulation of myofibroblasts with increased contractility [5], [7]. Therefore, we assessed myofibroblast and cell contractility associated genes in the cells. Strikingly, many of the genes in this category, including α-SMA, were markedly upregulated in SFBLs, although the cultures did not contain significant numbers of myofibroblasts. The high expression of these contractility-related genes is also functionally associated with increased contractility of SFBLs in the 3D cultures (unpublished data).

In order to find out whether intracellular signaling pathways involved in regulation of genes associated with scar formation are distinct between GFBLs and SFBLs in the 3D cultures, we assessed steady state activation of TGF-β, MAPK and β-catenin pathways. These pathways have been previously associated with fibrosis [43], [44]. Findings showed that only TGF-β signaling was significantly different between GFBLs and SFBLs. Therefore, we examined molecules involved in this pathway in more detail. TGF-βs are multifactorial growth factors that consist of three members, TGF-β1, -β2 and β3, in humans. Based on findings from scar-free fetal wound healing and *in vivo* experiments, the profibrotic effects of TGF-β1, and possibly TGF-β2, maybe balanced by anti-fibrotic TGF-β3 [3]. The findings showed that GFBLs expressed in general lower levels of TGFβ, especially TGF-β1 and TGF-β3, than SFBLs. Furthermore, molecules mediating the intracellular profibrotic TGF-β signaling, and previously associated with scar formation and fibrosis, including transcriptional (EGR1, EGR2 and EGR3), and translational positive regulator of this pathway (P311), were significantly higher in SFBLs [30]–[34]. Paradoxically, though, GFBLs expressed higher levels of TGF-β2, and 3D cultures contained elevated levels of total TGF-β1 protein, while SFBLs had higher levels of Cthrc1 and SMAD7 that suppress TGF-β signaling [35]–[37]. Therefore, regulation of autogenous TGF-β activity and signaling in the 3D cultures is likely complex, as also found *in vivo* [3], [69]. Accumulation of TGF-β in the ECM, and its biological activity, is modulated by ECM molecules (*e.g.* fibronectin, and fibrillin-1) that bind and store TGF-β as a latent molecule to the ECM. In addition, certain ECM molecules participate in TGF-β activation (*e.g*. thrombospondin-1) or inhibit its activity via binding to it (*e.g.* asporin, biglycan, decorin, fibromodulin, emilin-1 and emilin-3) [52], [56], [69], [70]. Interestingly, while there was no difference in the expression of EDA and EDB fibronectin isoforms, fibrillin-1, emilin isoforms and asporin, the expression of biglycan, decorin and fibromodulin was significantly elevated in SFBLs compared to GFBLs. At protein level, GFBLs secreted elevated levels of fibronectin and thrombospondin-1, while SFBLs produced significantly more biglycan and decorin. Thus, differential production of these TGF-β activating (thrombospondin-1) or scavenging (fibronectin, biglycan and decorin) ECM molecules cannot fully explain the contrasting findings that SFBLs expressed elevated TGF-β1 mRNA levels while higher TGF-β1 protein levels were found in GFBL cultures. Biologically active TGF-β1 has a short half-life as it becomes quickly internalized by TGF-β receptor-mediated endocytosis, followed by intracellular degradation in a process that can also at the same time trigger SMAD-mediated signaling [35], [71]. Therefore, it is tempting to speculate that lower levels of TGF-β1 protein despite of higher mRNA expression in SFBL compared to GFBL cultures may result from elevated usage (activation and endocytosis) of autogenous TGF-β1 by SFBLs, which could reduce the total amount of TGF-β1 in the cultures. This is supported by our findings that SFBLs showed significantly elevated expression of TGF-β receptors (TGF-βR1 and -βR2) that mediate TGF-β endocytosis and signaling, and a higher steady-state SMAD3 phosphorylation, the SMAD isoform that mediates the profibrotic TGF-β signaling [35]. This is similar to findings showing that scar forming skin wounds have higher numbers of pSMAD3 positive connective tissue cells compared to oral mucosal wounds [10]. Previous findings have also indicated that fibroblasts from skin have an increased expression of profibrotic genes as a response to TGF-β as compared to gingival fibroblasts [72]. Similarly, in the 3D culture model, increased pSMAD3 level in SFBLs was also associated with an increased expression of well known TGF-β regulated genes, including type I collagen, CCN2 (CTGF) and α-SMA. It is interesting to note that, while TGF-β2 expression was higher in GFBLs, unlike TGF-β1 and TGF-β3, TGF-β2 cannot be activated by cell-mediated traction forces because latent TGF-β2 lacks the RGD site recognized by integrins that mediate the activation [73]. Importantly, the traction forces-mediated pathway has been shown to be the main mechanism of TGF-β activation *in vivo* [74]. Thus, our findings suggest that SFBLs display an increased autogenous TGF-β signaling involving higher pSMAD3 levels, elevated expression of transcriptional and translational TGF-β signaling regulators, and TGF-β responsive genes. Whether the discovered significantly higher expression of contractility-associated proteins by SFBLs is linked with elevated autogenous TGF-β activation in the 3D cultures remains to be shown.

Taken together, the findings showed that GFBLs are phenotypically distinct from SFBLs when studied in the 3D ECM niche, the former expressing elevated levels of MMPs involved in regulation of inflammation and ECM remodeling, and the latter having higher expression of ECM molecules, TGF-β signaling and myofibroblast and cell contractility associated genes. These inherent differences in fibroblasts that participate in wound healing may underlie the ability of gingival wounds to heal faster and with reduced scar formation as compared to skin wounds.

## Supporting Information

Figure S1 **Comparison of gene expression in abdominal and breast skin SFBLs with GFBLs.** Out of the 28 genes analyzed, 27 genes showed similar expression pattern in both breast and abdominal SFBLs relative to GFBLs. Only one gene (MMP-2) was expressed at a markedly higher level by abdominal, but not by breast SFBLs, relative to GFBLs. Expression of mRNA was analyzed in 3D cultures 7 days post-seeding using three fibroblast lines from abdominal skin from different donors, and were compared with five parallel GFBL and breast SFBL lines (mean +/− SEM). Results for abdominal SFBLs are from two parallel experiments.
(TIF)

Table S1 **List of antibodies used for immunocytochemistry and Western blotting.**
(DOCX)

Table S2 **Primers used for real-time RT-PCR.**
(DOCX)

Table S3 **Real-time PCR analysis results for individual human breast skin and gingival fibroblast lines in seven-day 3D cultures.** Results show mean values relative to one GFBL line (GFBL-DC) obtained by the comparative Ct method. Results show all genes that displayed Ct<30 for at least one cell line. Expression of emilin-3, hevin-1, hevin-2 and MMP-13 was negligible (Ct = 30–34) in both GFBLs and SFBLs and are not shown. Cthrc1: Collagen triple helix containing-1; EGR1, -2, -3: Early Growth Response 1, -2, -3; FGF-2: Fibroblast Growth Factor-2; NAB1, -2: NGFI-A Binding Protein-1, -2; NMMIIA, -IIB: Non-Muscle Myosin IIA, -IIB; α-SMA: α-Smooth Muscle Actin; VEGF-α: TGF-βRI, -βRII: TGF-β Receptor 1, -2; Vascular Endothelial Growth Factor-α.
(DOCX)
