# Supplementary figures and images for: Human Gingival Fibroblasts Display a Non-Fibrotic Phenotype Distinct from Skin Fibroblasts in Three-Dimensional Cultures
Source: PLoS One. 2014 Mar 7;9(3):e90715. doi: 10.1371/journal.pone.0090715 (PMC3946595; doi:10.1371/journal.pone.0090715)

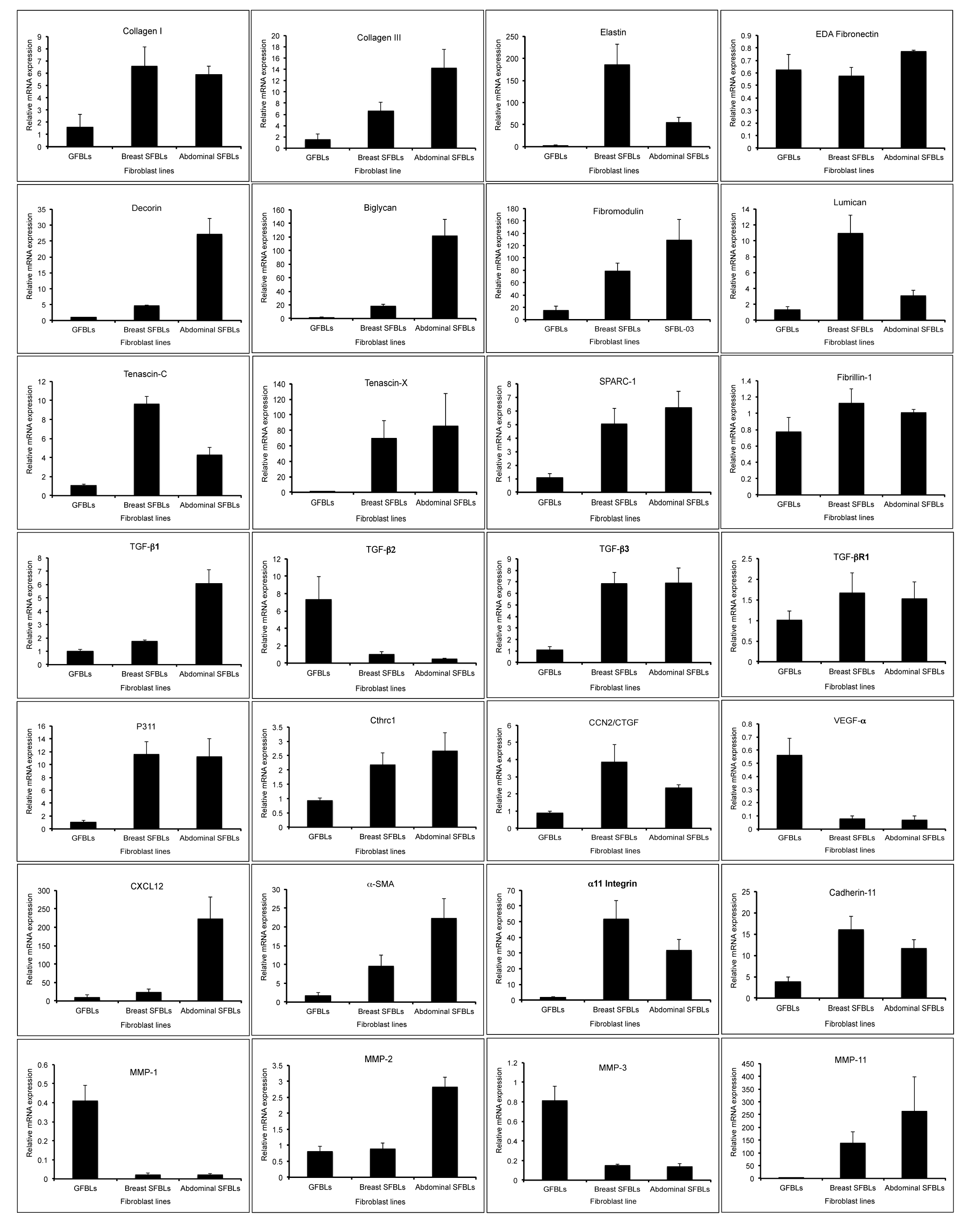

Supplement: Figure S1 — Comparison of gene expression in abdominal and breast skin SFBLs with GFBLs. Out of the 28 genes analyzed, 27 genes showed similar expression pattern in both breast and abdominal SFBLs relative to GFBLs. Only one gene (MMP-2) was expressed at a markedly higher level by abdominal, but not by breast SFBLs, relative to GFBLs. Expression of mRNA was analyzed in 3D cultures 7 days post-seeding using three fibroblast lines from abdominal skin from different donors, and were compared with five parallel GFBL and breast SFBL lines (mean +/− SEM). Results for abdominal SFBLs are from two parallel experiments. (TIF) [file pone.0090715.s001.tif]
