# Supplementary material for: Human Gingival Fibroblasts Display a Non-Fibrotic Phenotype Distinct from Skin Fibroblasts in Three-Dimensional Cultures
Source: PLoS One. 2014 Mar 7;9(3):e90715. doi: 10.1371/journal.pone.0090715 (PMC3946595; doi:10.1371/journal.pone.0090715)
Supplement: Table S1 — List of antibodies used for immunocytochemistry and Western blotting. (DOCX) [file pone.0090715.s002.docx]

**Supplemental Table S1. List of antibodies used for immunocytochemistry and Western blotting.** *Non-reducing conditions were used in Western blotting.

| **Antibody** | **Manufacturer** | **Source** | **Dilution** | |
| --- | --- | --- | --- | --- |
|  |  |  | **Immunostaining** | **Western blotting** |
| Anti-human Biglycan | Abnova Corp., Taipei, Taiwan | Mouse |  | 1:500 |
| Anti-human Decorin | R&D Systems Inc., Minneapolis, MN, USA | Mouse |  | 1:500 |
| Anti-human Fibromodulin (H-50) | Santa Cruz, Santa Cruz, CA, USA | Rabbit |  | 1:1000 |
| Anti-human Lumican (H-90) | Santa Cruz | Rabbit |  | 1:1000 |
| Anti-human Fibronectin-EDA | Abcam Inc., Cambridge, MA, USA | Mouse |  | 1:400 |
| Anti-Tenascin-C (BC-24) | Sigma | Mouse |  | 1:100* |
| Anti-Osteopontin  (MPIIIB101) | Developmental Studies Hybridoma Bank, Iowa, OH, USA | Mouse |  | 1:50 |
| Anti-SPARC-1 (H-90) | Santa Cruz | Rabbit |  | 1:200 |
| Anti-Thrombospondin-1 | Lab Vision Corp., Fremont, CA, USA | Mouse |  | 1:500* |
| Anti-Thrombospondin-2 (sc-136238) | Santa Cruz | Mouse |  | 1:200 |
| Anti-human α-SMA | Sigma, St. Louis, MO, USA | Mouse | 1:200 | 1:500 |
| Anti-β-Tubulin (ab21057) | Abcam Inc. | Goat | 1:1000 | 1:5000 |
| Anti-human β-Actin  (ab8227) | Abcam Inc. | Rabbit | 1:1000 | 1:5000 |
| Anti-MMP-1 (N-17) (sc-8834-R) | Santa Cruz | Rabbit |  | 1:1000 |
| Anti-MMP-3 (ab77962) | Abcam Inc. | Mouse |  | 1:2000 |
| Anti-MMP-10 | R&D Systems Inc. | Goat |  | 1:1000 |
| Anti-TGF-β1 pAb | Promega, Madison, WI, USA | Rabbit |  | 1:500 |
| Anti-VEGF (A-20)  (sc-152) | Santa Cruz | Rabbit |  | 1:500* |
| Anti-human SMAD3 (ab28379) | Abcam Inc. | Rabbit |  | 1:2000 |
| Anti-human phospho-SMAD3 (ab52903) | Abcam Inc. | Rabbit |  | 1:2000 |
