# Supplementary material for: Human Gingival Fibroblasts Display a Non-Fibrotic Phenotype Distinct from Skin Fibroblasts in Three-Dimensional Cultures
Source: PLoS One. 2014 Mar 7;9(3):e90715. doi: 10.1371/journal.pone.0090715 (PMC3946595; doi:10.1371/journal.pone.0090715)
Supplement: Table S2 — Primers used for real-time RT-PCR. (DOCX) [file pone.0090715.s003.docx]

**Supplemental Table S2. Primers used for real-time RT-PCR.**

| **GeneBank** | **Gene** | **Primer sequence** | **Orientation** | **Location** | **Amplicon (bp)** |
| --- | --- | --- | --- | --- | --- |
| **Structural ECM Proteins** | | | | | |
| BC036531 | Collagen type I (alpha 1) | AACCAAGGCTGCAACCTGGA | Forward | 3951-3970 | 80 |
|  |  | GGCTGAGTAGGGTACACGCAGG | Reverse | 4030-4009 |  |
| NM_000090 | Collagen type III (alpha 1) | CTCCTGGGATTAATGGTAGT | Forward | 1271-1290 | 70 |
|  |  | CCAGGAGCTCCAGGAAT | Reverse | 1340-1324 |  |
| NM_000501 | Elastin | GGGTCCCTGGGGCCATTC | Forward | 174-191 | 62 |
|  |  | CAGGCCCCAGCGCTGGAT | Reverse | 236-219 |  |
| NM_000138 | Fibrillin-1 | AGGAAACGGAGAAGCACAA | Forward | 8577-8595 | 103 |
|  |  | CTGTCTTCTCAACATCCCAA | Reverse | 8679-8660 |  |
| NM_007046 | Emilin-1 | CCTTCGACAGAGTCCTGCTCAA | Forward | 3162-3183 | 65 |
|  |  | CGCTGTGAACACGCCTGTC | Reverse | 3226-3208 |  |
| NM_032048 | Emilin-2 | GTGAACATGGCCACTGACTT | Forward | 3513-3532 | 87 |
|  |  | GCCCTCAGAGTGTAGATACAG | Reverse | 3600-3580 |  |
| NM_052846 | Emilin-3 | TCCCTTAGACGAGATCCTAAGCA | Forward | 962-984 | 72 |
|  |  | TCTAGAAGCTGCACCTTGGTC | Reverse | 1033-1013 |  |
| NM_212482 | EDA-FN (EDA-fibronectin) | CACAGTCAGTGTGGTTGCCT | Forward | 5633-5652 | 68 |
|  |  | CTGTGGACTGGGTTCCAATCA | Reverse | 5700-5680 |  |
| NM_212482 | EDB-FN (EDB-fibronectin) | CAGTAGTTGCGGCAGGAGAA | Forward | 4168-4188 | 65 |
|  |  | GTATCCTACTGAGGAGTCCACAAAATC | Reverse | 4232-4206 |  |
| **Matricellular Proteins** | | | | | |
| NM_003246 | Thrombospondin-1   (THBS-1) | CATCTTGTTCTGTGACATGTGG | Forward | 1513-1534 | 92 |
|  |  | TTCACAGGGTTTCCCGTTC | Reverse | 1604-1586 |  |
| NM_003247 | Thrombospondin-2   (THBS-2) | TTGTGTTCAACCCAGACCA | Forward | 2989-3007 | 107 |
|  |  | CAGGACACACATCATCAATATC | Reverse | 3096-3075 |  |
| NM_003118 | SPARC-1 | AGCAAGAAGCCCTGCCTGA | Forward | 164-182 | 101 |
|  |  | TCCTACTTCCACCTGGACAGGATT | Reverse | 264-241 |  |
| NM_002160 | Tenascin-C | CAACCTGATGGGGAGATATGGGGA | Forward | 6769-6792 | 75 |
|  |  | GAGTGTTCGTGGCCCTTCCAG | Reverse | 6846-6826 |  |
| NM_019105 | Tenascin-X | CCTGGAGCCAGACCATAAATACAAG | Forward | 6334-6358 | 57 |
|  |  | CGCTGGCCACCGTGGAA | Reverse | 6390-6374 |  |
| NM_000582 | Osteopontin | CAGTTGCAGCCTTCTCAG | Forward | 118-136 | 82 |
|  |  | CTAGGAGGCAAAAGCAAATC | Reverse | 199-180 |  |
| NM_006475 | Periostin | ACACGAGAAGAACGAATCAT | Forward | 199-181 | 97 |
|  |  | GTAACAATTTCTTCAGAGTTTCTTC | Reverse | 199-182 |  |
| NM_001554 | CCN1 | CATGATGATCCAGTCCTGCAA | Forward | 199-185 | 86 |
|  |  | TGTCATTGAACAGCCTGTAGAAG | Reverse | 199-186 |  |
| NM_001901 | CCN2/CTGF | ATGATGTTCATCAAGACCTGTGCCTG | Forward | 199-183 | 80 |
|  |  | CTTCCTGTAGTACAGGGATTCAAAGATGTC | Reverse | 199-184 |  |
| NM_002514 | CCN3 | CTCAGATCTGGAGCCATGCGA | Forward | 199-187 | 85 |
|  |  | CGTGCAGATGCCAGTCTGGTTG | Reverse | 199-188 |  |
| NM_001128310 | Hevin-1 | CTGCAGCTGCAATCCCGA | Forward | 199-189 | 115 |
|  |  | TCTTCAGCTTCAGCCCTTAAACT | Reverse | 199-190 |  |
| NM_001128310 | Hevin-2 | CCAGCATCCTCCTCTGTTC | Forward | 199-191 | 132 |
|  |  | TGGTGCATAGGTTGTTGTCA | Reverse | 199-192 |  |
| **Small Leucine-Rich Proteoglycans** | | | | | |
| NM_001711 | BGN (Biglycan) | CTCAAGCTCCTCCAGGTGGTC | Forward | 1067-1087 | 93 |
|  |  | CCGAAGCCCATGGGACAGAAGTC | Reverse | 1151-1127 |  |
| BT019800 | DCN (Decorin) | CTGACACAACTCTGCTAGAC | Forward | 242-261 | 97 |
|  |  | GACAAGAATCAATGCGTGAAG | Reverse | 339-319 |  |
| NM_002023 | FMOD (Fibromodulin) | CACAATGAGATCCAGGAAG | Forward | 761-779 | 85 |
|  |  | TCCGAAGGTGGTTATAACTC | Reverse | 845-826 |  |
| BT006707 | LUM (Lumican) | TAGACAACAATAAGATCAGCAACA | Forward | 635-658 | 85 |
|  |  | TTCGTTGTGAGATAAACGCAG | Reverse | 720-700 |  |
| NM_017680 | ASPN (Asporin) | CCTCCAGATAATCTTCCTTCATTCTA | Forward | 1183-1213 | 81 |
|  |  | CTTCATCTTTGGCACTGTTGG | Reverse | 1244-1264 |  |
| **TGF-β Signaling** | | | | | |
| NM_000660 | TGF-β1 (Transforming Growth Factor-β1) | CAACGAAATCTATGACAAGTTCAAGCAG | Forward | 1218-1245 | 76 |
|  |  | CTTCTCGGAGCTCTGATGTG | Reverse | 1294-1275 |  |
| NM_003238 | TGF-β2 (Transforming Growth Factor-β2) | TGGTGAAAGCAGAGTTCAGAG | Forward | 1883-1903 | 140 |
|  |  | CACAACTTTGCTGTCGATGTAG | Reverse | 2022-2001 |  |
| NM_003239 | TGF-β3 (Transforming Growth Factor-β3) | ACACCAATTACTGCTTCCGCAA | Forward | 1161-1182 | 81 |
|  |  | GCCTAGATCCTGTCGGAAGTC | Reverse | 1242-1220 |  |
| NM_001130916 | TGF-βR1 (TGF-β Receptor 1) | GTGTATAGCTGAAATTGACTTAA | Forward | 286-308 | 99 |
|  |  | TGATTGCAGCAATATGTTGTA | Reverse | 384-364 |  |
| NM_003242 | TGF-βR2 (TGF-β Receptor 2) | CTGGTGAGACTTTCTTCATGTG | Forward | 768-789 | 127 |
|  |  | CTGATGCCTGTCACTTGAAA | Reverse | 894-875 |  |
| NM_001190823 | SMAD7 | ATTCCCAACTTCTTCTGGAG | Forward | 328-347 | 96 |
|  |  | TGGACACAGTAGAGCCTC | Reverse | 424-407 |  |
| NM_138455 | Cthrc-1 (Collagen triple helix containing-1) | TGGAAGGACTTTGTGAAGGA | Forward | 689-708 | 104 |
|  |  | AATTCCATCCAGTAGAAGCATC | Reverse | 793-772 |  |
| NM_001142483 | P311 | CCTGGACTGAAGAGAGG | Forward | 321-447 | 78 |
|  |  | CAGACAAAGAGTTCTGGGTA | Reverse | 508-489 |  |
| NM_001964 | EGR1 (Early Growth Response 1) | ACGTCTTGGTGCCTTTTGTG | Forward | 2663-2682 | 75 |
|  |  | GAGGTGAGCATGTCCCTCA | Reverse | 2737-2719 |  |
| NM_001136179 | EGR2 (Early Growth Response 2) | AGCTTTGCTCCCGTCTCTG | Forward | 469-487 | 88 |
|  |  | AGCTGGCACCAGGGTACT | Reverse | 556-539 |  |
| NM_004430 | EGR3 (Early Growth Response 3) | CGTTGGACAGCAATCTCTTC | Forward | 903-922 | 74 |
|  |  | AATGGAGCCCATGTCGTTG | Reverse | 976-958 |  |
| NM_005966 | NAB1 (NGFI-A Binding Protein-1) | CAAAGTCCCACTCATCAGAGA | Forward | 1930-1950 | 114 |
|  |  | TCACAGCTATCTTGAATCTTCAG | Reverse | 2043-2020 |  |
| NM_005967 | NAB2 (NGFI-A Binding Protein-2) | CACATCCCTGCTAAAGCTGAA | Forward | 1170-1190 | 111 |
|  |  | ATGATGCTGTATTTGCGGATCT | Reverse | 1280-1259 |  |
| **Angiogenesis** | | | | | |
| NM_001171630 | VEGF-α (Vascular Endothelial Growth Factor-α) | AGTGTGTGCCCACTGAGGA | Forward | 1316-1334 | 97 |
|  |  | GTGCTGTAGGAAGCTCATCTC | Reverse | 1413-1393 |  |
| NM_002006 | FGF-2 (Fibroblast Growth Factor-2) | AGTTGGTATGTGGCACTGAA | Forward | 831-850 | 75 |
|  |  | GTATAGCTTTCTGCCCAGGTC | Reverse | 886-906 |  |
| NM_199168 | CXCL12/SDF-1α | TACAGATGCCCATGCCGA | Forward | 174-191 | 93 |
|  |  | CTGAAGGGCACAGTTTGGAG | Reverse | 266~247 |  |
| **Myofibroblast- and Cell Contractility-Associated Genes** | | | | | |
| NM_001613 | α –SMA (α-Smooth Muscle Actin) | AGCGTGGCTATTCCTTCGT | Forward | 637-655 | 97 |
|  |  | CTCATTTTCAAAGTCCAGAGCTACA | Reverse | 733-707 |  |
| NM_001004439 | α11 Integrin | GAAGGCACCAACAAGAACGA | Forward | 1144-1163 | 60 |
|  |  | AGGAAAAGCCCGTCTGTGA | Reverse | 1204-1186 |  |
| NM_001792 | Cadherin-2 | TGAGGAGTCAGTGAAGGAG | Forward | 843-861 | 91 |
|  |  | CTTCTGCCTTTGTAGGTGG | Reverse | 933-915 |  |
| NM_001797 | Cadherin-11 | TTCCTTCGTGTTGTCATTTGTTG | Forward | 174-196 | 89 |
|  |  | CTTCTTCACCCATTGGATACTTG | Reverse | 262-240 |  |
| NM_002473 | NMMIIA (Non-muscle Myosin IIA) | ACCGAGAAGATCAATCCATC | Forward | 722-741 | 81 |
|  |  | AGATACTGGATGACCTTCTTG | Reverse | 803-783 |  |
| NM_005964 | NMMIIB (Non-muscle Myosin IIB) | CCGTTTTACATAATCTGAAGGATC | Forward | 395-418 | 98 |
|  |  | TTGGAAGATTCTTGTAAGGGTT | Reverse | 493-472 |  |
| NM_001101 | β-Actin | AGGCCCAGAGCAAGAGA | Forward | 254-270 | 72 |
|  |  | CAGTTGGTGACGATGCCG | Reverse | 320-303 |  |
| NM_001614 | ϒ-Actin | GTCGCAATGGAAGAAGAGATCG | Forward | 134-155 | 85 |
|  |  | AGCGTCGTCCCCAGCAAAA | Reverse | 218-199 |  |
| **Intracellular ECM Degradation-Associated Genes** | | | | | |
| NM_000396 | CTSK (Cathepsin K) | TCGACTATCGAAAGAAAGGATA | Forward | 585-606 | 70 |
|  |  | AAAGCCCAACAGGAACCA | Reverse | 655-637 |  |
| AF134838 | Endo180 (CD280) | AAGAGGCCCAGCTGGTCA | Forward | 3144-3161 | 100 |
|  |  | GCATGGAGGCCAATCCAAAG | Reverse | 3243-3224 |  |
| NM_002332 | LRP-1 (Low Density Lipoprotein Receptor-Related Protein-1) | ACGCCTCTGACGTGGTCCT | Forward | 12861-12879 | 73 |
|  |  | CATTTCTTGCGGTCACATGGGTTG | Reverse | 12933-12910 |  |
| **MMPs and their Inhibitors** | | | | | |
| NM_002421 | MMP-1 | GCTAACAAATACTGGAGGTATGATG | Forward | 1250-1275 | 100 |
|  |  | GTCATGTGCTATCATTTTGGGA | Reverse | 1304-1325 |  |
| NM_001127891 | MMP-2 | AGACATACATCTTTGCTGGAG | Forward | 1900-1920 | 88 |
|  |  | ATCTGCGATGAGCTTGG | Reverse | 1988-1972 |  |
| NM_001166308 | MMP-3 | ATGATGAACAATGGACAAAGGA | Forward | 661-682 | 91 |
|  |  | GAGTGAAAGAGACCCAGGGA | Reverse | 751-732 |  |
| NM_214207 | MMP-7 | TATGCTGCAACTCATGAAC | Forward | 722-740 | 82 |
|  |  | CGTAGGTTGGATACATCAC | Reverse | 804-785 |  |
| NM_002425 | MMP-10 | TTATACACCAGATTTGCCAAGA | Forward | 394-415 | 56 |
|  |  | TTCAGAGCTTTCTCAATGG | Reverse | 450-432 |  |
| NM_005940 | MMP-11 | CTATCCTCCAAAGCCATTGTAA | Forward | 2258-2179 | 106 |
|  |  | CAACTGTGTTTAATGACAATCCTC | Reverse | 2254-2231 |  |
| NM_002426 | MMP-12 | GTATGATGAAAGGAGACAGATGAT | Forward | 1303-1326 | 166 |
|  |  | TACGTTGGAGTAGGAAGTCAT | Reverse | 1469-1449 |  |
| NM_002427 | MMP-13 | CAGGAATTGGTGATAAAGTAGAT | Forward | 1302-1324 | 85 |
|  |  | CTGTATTCAAACTGTATGGGTC | Reverse | 1365-1386 |  |
| NM_003254 | TIMP-1 | CTGTGTCCCACCCCACC | Forward | 267-283 | 64 |
|  |  | GAACTTGGCCCTGATGACGA | Reverse | 330-311 |  |
| NM_003255 | TIMP-2 | ACATTTATGGCAACCCTATCAA | Forward | 481-502 | 70 |
|  |  | TCAGGCCCTTTGAACATCTTTA | Reverse | 550-529 |  |
| NM_000362 | TIMP-3 | AGGACGCCTTCTGCAAC | Forward | 1281-1297 | 68 |
|  |  | CTCCTTTACCAGCTTCTTCC | Reverse | 1348-1329 |  |
| NM_003256 | TIMP-4 | ACCTGTCCTTGGTGCAGA | Forward | 927-944 | 80 |
|  |  | TGTAGCAGGTGGTGATTTGG | Reverse | 1004-985 |  |
| **Housekeeping Genes** | | | | | |
| NM_002046 | GAPDH | CTTTGTCAAGCTCATTTCCTGGTA | Forward | 1020-1043 | 70 |
|  |  | GGCCATGAGGTCCACCA | Reverse | 1089-1073 |  |
| M31642 | HPRT1 | TGTTGGATTTGAAATTCCAGACAAG | Forward | 619-643 | 107 |
|  |  | CTTTTCCAGTTTCACTAATGACACAA | Reverse | 727-700 |  |
| NM_021009 | UBC | GTGGCACAGCTAGTTCCGT | Forward | 371-389 | 96 |
|  |  | CTTCACGAAGATCTGCATTGTCA | Reverse | 444-467 |  |
| NM_004048 | B2M | TGTCTTTCAGCAAGGACTGGTCTTTC | Forward | 281-306 | 92 |
|  |  | ATGGTTCACACGGCAGGCATA | Reverse | 351-372 |  |
| BC009255 | ALG9 | GAATGACCAGAATCTAGAAGAGCCA | Forward | 1668-1692 | 82 |
|  |  | TCTCATGGTGTCCAAATCCACTAAA | Reverse | 1749-1725 |  |
