# Supplementary material for: Human Gingival Fibroblasts Display a Non-Fibrotic Phenotype Distinct from Skin Fibroblasts in Three-Dimensional Cultures
Source: PLoS One. 2014 Mar 7;9(3):e90715. doi: 10.1371/journal.pone.0090715 (PMC3946595; doi:10.1371/journal.pone.0090715)
Supplement: Table S3 — Real-time PCR analysis results for individual human breast skin and gingival fibroblast lines in seven-day 3D cultures. Results show mean values relative to one GFBL line (GFBL-DC) obtained by the comparative Ct method. Results show all genes that displayed Ct<30 for at least one cell line. Expression of emilin-3, hevin-1, hevin-2 and MMP-13 was negligible (Ct = 30–34) in both GFBLs and SFBLs and are not shown. Cthrc1: Collagen triple helix containing-1; EGR1, -2, -3: Early Growth Response 1, -2, -3; FGF-2: Fibroblast Growth Factor-2; NAB1, -2: NGFI-A Binding Protein-1, -2; NMMIIA, -IIB: Non-Muscle Myosin IIA, -IIB; α-SMA: α-Smooth Muscle Actin; VEGF-α: TGF-βRI, -βRII: TGF-β Receptor 1, -2; Vascular Endothelial Growth Factor-α. (DOCX) [file pone.0090715.s004.docx]

**Supplemental Table S3. Real-time PCR analysis results for individual human breast skin and gingival fibroblast lines in seven-day 3D cultures.**

Results show mean values relative to one GFBL line (GFBL-DC) obtained by the comparative Ct method. Results show all genes that displayed Ct<30 for at least one cell line. Expression of Emilin-3, Hevin-1, Hevin-2 and MMP-13 was negligible (Ct=30-34) in both GFBLs and SFBLs and are not shown. Cthrc1: Collagen triple helix containing-1; EGR1, -2, -3: Early Growth Response 1, -2, -3; FGF-2: Fibroblast Growth Factor-2; NAB1, -2: NGFI-A Binding Protein-1, -2; NMMIIA, -IIB: Non-Muscle Myosin IIA, -IIB; α-SMA: α-Smooth Muscle Actin; VEGF-α: TGF-βRI, -βRII: TGF-β Receptor 1, -2; Vascular Endothelial Growth Factor-α.

|  | **Breast Skin Fibroblasts** | | | | | **Gingival Fibroblasts** | | | | |
| --- | --- | --- | --- | --- | --- | --- | --- | --- | --- | --- |
| **Gene** | **SFBL-1-2** | **SFBL-2-C** | **SFBL-4-1** | **SFBL-302** | **SFBL-406** | **GFBL-DC** | **GFBL-DW** | **GFBL-HN** | **GFBL-OL** | **GFBL-IE** |
| γ-actin | 0.65 | 1.43 | 1.04 | 0.84 | 1.32 | 1.00 | 1.01 | 0.88 | 0.60 | 1.22 |
| β-actin | 1.48 | 2.08 | 1.31 | 0.88 | 1.96 | 1.00 | 1.49 | 0.88 | 0.89 | 1.22 |
| Asporin | 2.29 | 1.39 | 0.76 | 0.20 | 1.37 | 1.00 | 0.15 | 0.94 | 0.08 | 0.11 |
| Biglycan | 15.73 | 26.45 | 23.50 | 11.13 | 14.64 | 1.00 | 0.21 | 0.30 | 3.17 | 4.07 |
| Cadherin-2 | 7.60 | 7.79 | 4.75 | 4.41 | 7.88 | 1.00 | 1.26 | 0.25 | 1.60 | 1.49 |
| Cadherin-11 | 13.21 | 27.19 | 17.32 | 7.22 | 14.97 | 1.00 | 7.73 | 4.29 | 2.29 | 3.84 |
| Cathepsin K | 2.64 | 3.84 | 3.59 | 3.83 | 2.80 | 1.00 | 1.06 | 1.89 | 0.86 | 1.50 |
| CCN1 | 0.96 | 0.99 | 0.89 | 0.06 | 0.40 | 1.00 | 1.07 | 0.89 | 0.73 | 0.09 |
| CCN2/CTGF | 5.11 | 5.93 | 2.65 | 0.54 | 5.13 | 1.00 | 0.93 | 1.26 | 0.68 | 0.53 |
| CCN3 | 0.94 | 1.08 | 2.11 | 1.00 | 2.19 | 1.00 | 1.13 | 2.04 | 1.30 | 0.85 |
| Collagen I | 10.07 | 8.57 | 9.13 | 1.73 | 3.39 | 1.00 | 0.21 | 0.57 | 5.77 | 0.41 |
| Collagen III | 35.56 | 12.22 | 28.76 | 11.39 | 23.67 | 1.00 | 0.49 | 4.27 | 0.57 | 1.25 |
| Cthrc1 | 1.51 | 2.32 | 3.226 | 1.01 | 2.90 | 1.00 | 0.70 | 0.957 | 1.09 | 0.98 |
| CXCL12/SDF-1α | 18.2 | 34.93 | 49.22 | 7.79 | 9.80 | 1.00 | 2.85 | 35.3 | 1.32 | 3.96 |
| Decorin | 4.58 | 3.98 | 5.53 | 4.71 | 4.32 | 1.00 | 0.81 | 0.64 | 1.27 | 1.35 |
| EGR1 | 2.76 | 1.87 | 2.30 | 0.31 | 0.69 | 1.00 | 1.11 | 0.19 | 0.15 | 0.30 |
| EGR2 | 5.19 | 2.11 | 1.76 | 1.07 | 5.02 | 1.00 | 0.48 | 0.44 | 0.19 | 0.12 |
| EGR3 | 63.37 | 64.01 | 56.72 | 18.81 | 75.72 | 1.00 | 18.4 | 2.57 | 2.24 | 1.07 |
| Elastin | 315.81 | 234.26 | 193.51 | 30.92 | 153.48 | 1.00 | 3.78 | 2.36 | 0.50 | 6.89 |
| Emilin-1 | 1.62 | 1.59 | 2.61 | 1.54 | 3.03 | 1.00 | 0.50 | 1.66 | 1.27 | 3.15 |
| Emilin-2 | 1.34 | 4.19 | 6.36 | 2.68 | 3.11 | 1.00 | 0.29 | 1.22 | 0.74 | 4.55 |
| Endo180 (CD280) | 2.93 | 2.67 | 2.51 | 1.705 | 1.71 | 1.00 | 0.59 | 2.00 | 1.10 | 2.15 |
| FGF-2 | 0.88 | 0.76 | 0.73 | 0.28 | 0.49 | 1.00 | 1.18 | 0.98 | 0.59 | 0.37 |
| Fibrillin-1 | 1.10 | 1.11 | 1.56 | 0.52 | 1.34 | 1.00 | 0.30 | 1.19 | 0.87 | 0.50 |
| Fibromodulin | 70.86 | 44.41 | 90.54 | 116.32 | 74.60 | 1.00 | 17.98 | 2.12 | 15.83 | 38.47 |
| Fibronectin EDA | 0.68 | 0.64 | 0.63 | 0.30 | 0.62 | 1.00 | 0.77 | 0.74 | 0.40 | 0.21 |
| Fibronectin EDB | 0.50 | 0.59 | 0.52 | 0.12 | 0.26 | 1.00 | 0.86 | 0.61 | 0.38 | 0.11 |
| Integrin α11 | 60.1 | 81.84 | 53.17 | 9.79 | 53.17 | 1.00 | 2.58 | 3.68 | 0.24 | 0.44 |
| LRP-1 | 1.00 | 1.14 | 1.40 | 0.21 | 0.37 | 1.00 | 0.72 | 0.90 | 0.90 | 0.38 |
| Lumican | 14.52 | 15.93 | 13.29 | 5.39 | 5.39 | 1.00 | 0.35 | 0.60 | 2.32 | 2.29 |
| MMP-1 | 0.03 | 0.01 | 0.02 | 0.04 | 0.02 | 1.00 | 0.03 | 0.25 | 0.48 | 0.30 |
| MMP-2 | 1.15 | 1.06 | 1.28 | 0.41 | 0.58 | 1.00 | 0.73 | 1.23 | 0.69 | 0.41 |
| MMP-3 | 0.17 | 0.19 | 0.13 | 0.13 | 0.11 | 1.00 | 0.17 | 0.85 | 0.48 | 0.13 |
| MMP-7 | 2.12 | 0.86 | 1.69 | 1.24 | 1.14 | 1.00 | 0.14 | 0.46 | 0.16 | 0.24 |
| MMP-10 | 0.03 | 0.013 | 0.019 | 0.04 | 0.03 | 1.00 | 0.037 | 0.26 | 0.48 | 0.32 |
| MMP-11 | 117.97 | 57.325 | 45.74 | 282.00 | 191.46 | 1.00 | 0.95 | 1.54 | 3.97 | 4.00 |
| MMP-12 | 0.21 | 1.17 | 1.11 | 0.374 | 0.283 | 1.00 | 0.22 | 0.36 | 1.18 | 0.45 |
| NAB1 | 0.74 | 2.24 | 0.94 | 0.51 | 1.04 | 1.00 | 1.49 | 1.00 | 0.98 | 0.92 |
| NAB2 | 1.74 | 1.77 | 1.66 | 1.40 | 1.82 | 1.00 | 1.64 | 1.45 | 0.80 | 1.95 |
| NMMIIA | 1.44 | 1.95 | 1.84 | 0.76 | 1.84 | 1.00 | 1.04 | 0.77 | 0.54 | 0.88 |
| NMMIIB | 3.81 | 6.21 | 4.01 | 2.68 | 3.38 | 1.00 | 1.8 | 0.63 | 1.28 | 1.56 |
| Osteopontin | 1.20 | 3.61 | 2.19 | 1.32 | 1.34 | 1.00 | 0.45 | 0.43 | 0.66 | 0.34 |
| P311 | 16.87 | 13.87 | 11.15 | 4.86 | 11.23 | 1.00 | 0.48 | 0.82 | 0.81 | 2.23 |
| Periostin | 3.14 | 3.17 | 0.60 | 0.82 | 2.60 | 1.00 | 0.03 | 1.74 | 1.25 | 0.19 |
| SMAD7 | 3.43 | 3.13 | 2.45 | 1.20 | 2.31 | 1.00 | 1.27 | 1.21 | 0.61 | 1.29 |
| α-SMA | 10.93 | 17.35 | 4.59 | 1.05 | 13.51 | 1.00 | 4.65 | 0.84 | 1.48 | 0.92 |
| SPARC1 | 8.07 | 6.99 | 6.18 | 1.37 | 4.91 | 1.00 | 0.91 | 2.15 | 0.56 | 0.79 |
| TGF-β1 | 1.82 | 1.50 | 1.55 | 1.98 | 1.94 | 1.00 | 0.88 | 0.87 | 0.84 | 1.49 |
| TGF-β2 | 0.81 | 0.94 | 0.46 | 0.82 | 2.15 | 1.00 | 5.30 | 15.88 | 3.77 | 10.5 |
| TGF-β3 | 6.54 | 8.02 | 7.03 | 3.47 | 9.22 | 1.00 | 0.72 | 0.68 | 0.79 | 2.23 |
| TGF-βR1 | 1.08 | 3.59 | 1.37 | 1.23 | 1.07 | 1.00 | 1.84 | 1.00 | 0.59 | 0.58 |
| TGF-βR2 | 1.51 | 2.80 | 1.90 | 1.01 | 1.72 | 1.00 | 1.45 | 1.04 | 0.99 | 1.45 |
| Thrombospondin-1 | 1.38 | 1.69 | 1.71 | 0.68 | 1.25 | 1.00 | 0.57 | 1.63 | 0.36 | 0.30 |
| Thrombospondin-2 | 3.32 | 2.79 | 2.63 | 1.24 | 1.84 | 1.00 | 0.26 | 2 | 0.3 | 0.53 |
| TIMP1 | 0.84 | 0.87 | 1.05 | 0.53 | 0.74 | 1.00 | 2.37 | 0.8 | 0.81 | 0.64 |
| TIMP2 | 1.98 | 1.77 | 1.37 | 0.76 | 0.98 | 1.00 | 0.67 | 1.13 | 0.87 | 0.71 |
| TIMP3 | 1.36 | 1.3 | 1.4 | 0.57 | 1.42 | 1.00 | 0.45 | 2.54 | 0.25 | 0.32 |
| TIMP4 | 0.28 | 0.27 | 0.65 | 0.28 | 0.32 | 1.00 | 3.59 | 2.27 | 2.17 | 1.22 |
| Tenascin-C | 9.48 | 8.47 | 11.26 | 11.34 | 7.69 | 1.00 | 0.68 | 1.17 | 1.73 | 0.92 |
| Tenascin-X | 81.1 | 48.77 | 153.54 | 41.74 | 22.6 | 1.00 | 0.78 | 4.25 | 1.97 | 1.03 |
| VEGF-α | 0.13 | 0.12 | 0.09 | 0.02 | 0.04 | 1.00 | 0.61 | 0.68 | 0.46 | 0.05 |
